# Deep Prior Framework: integrating functional specificity with general plausibility for targeted protein evolution

**DOI:** 10.1093/bib/bbag279

**Published:** 2026-06-11

**Authors:** Senxin Zhang, Yining Qin, Hanwen Zhu, Feilong Meng, Lei Jia, Xiaoqi Zheng

**Affiliations:** Department of Mathematics, Shanghai Normal University, No. 100 Guilin Road, Xuhui District, Shanghai 200234, China; Key Laboratory of RNA Innovation, Science and Engineering, Shanghai Academy of Natural Sciences (SANS), Shanghai Institute of Biochemistry and Cell Biology, Center for Excellence in Molecular Cell Science, Chinese Academy of Sciences, University of Chinese Academy of Sciences, No. 320 Yueyang Road, Xuhui District, Shanghai 200031, China; The Guangxi Key Laboratory of Intelligent Precision Medicine, 25 East Section of Gaoxin Avenue, High-tech District, Guangxi Zhuang Autonomous Region, Nanning 530007, China; Department of Biomedical Informatics, School of Basic Medical Sciences, Peking University Health Science Center, No. 38 Xueyuan Road, Haidian District, Beijing 100191, China; Key Laboratory of RNA Innovation, Science and Engineering, Shanghai Academy of Natural Sciences (SANS), Shanghai Institute of Biochemistry and Cell Biology, Center for Excellence in Molecular Cell Science, Chinese Academy of Sciences, University of Chinese Academy of Sciences, No. 320 Yueyang Road, Xuhui District, Shanghai 200031, China; Biomaterials and Stem Cell Research Laboratory, Suzhou Institute of Biomedical Engineering and Technology, Chinese Academy of Science, No. 88 Keling Road, High-tech District, Suzhou 215011, China; Center for Single-Cell Omics, School of Public Health, Shanghai Jiao Tong University School of Medicine, No. 1 Banxia Road, Pudong District, Shanghai 201318, China; Hainan International Medical Center, Shanghai Jiao Tong University School of Medicine, No. 1 Guangci Road, Qiongha 571400, Hainan, China

**Keywords:** protein evolution, protein language models, functional specificity, machine learning, UniProtKB

## Abstract

The efficiency of directed protein evolution largely relies on computational methods to enrich mutants with high fitness. Traditional strategies, such as zero-shot approaches based on Protein Language Models (PLMs), primarily leverage general “plausibility” priors learned from natural sequences. However, in the absence of experimental feedback, their ability to guide evolution toward specific functions (“specificity”) remains limited. Here, we introduce the Deep Prior Framework (DPF), a novel paradigm that integrates universal structural plausibility with task-oriented specificity priors. DPF incorporates an innovative Bernoulli-Attention (BATT) module within a Mixture of Experts architecture, enabling efficient screening of high-fitness mutants. Benchmarking on nine deep mutational scanning datasets, DPF outperforms existing methods in terms of PLM-based method. More importantly, our method also shows high performance on *in silico* directed evolution of *Blastobotrys adeninivorans* xanthine dehydrogenase (*Ba*XD) without intermediate experimental feedback. Experimental validation of the top-ranked mutants showed an average activity enhancement of over four-fold compared with WT, with the best mutant achieving more than a nine-fold improvement. Furthermore, we applied DPF to a large-scale annotation of unreviewed sequences in UniProt Knowledgebase (UniProtKB). Of the 42 913 366 predicted samples (~21.56% of the total), 90.07% (38 934 581 proteins) were assigned high-confidence functional labels. In summary, this study demonstrates that DPF, by incorporating specificity-aware functional priors, can significantly advance efficient and targeted protein engineering.

## Introduction

Natural protein evolution is a process that is straightforward in principle yet challenging in practice. Within a vast pool of potential variants, only a tiny fraction of mutants exhibit beneficial fitness effects [1, 2]. This inherently stochastic exploration process, while universal, is highly inefficient. Relying solely on high-throughput experimental screening to navigate this immense space presents an insurmountable burden [3–5]. Therefore, computational methods capable of enriching for high-fitness candidates are of critical importance.

We argue that the fitness of a protein can be deconstructed into two orthogonal dimensions: plausibility [6], which represents the universal physicochemical constraints that all functional proteins must satisfy, and specificity [7, 8], which entails the personalized optimizations required for a particular biological function (e.g. DNA-binding capability). Both dimensions can be viewed as forms of prior knowledge. Logically, the more accurately a method captures these priors, the more effectively it can guide the discovery of mutants with higher fitness.

Early directed evolution techniques, such as error-prone PCR—a method that mimics the natural random process by introducing biased randomness [9–11]. Their mutational spectra are constrained by biochemical factors like polymerase specificity, forming an intrinsic statistical prior that is agnostic to the target function. While this prior imparts a degree of structure to the search space, the absence of targeted guidance based on protein structure or function ultimately limits exploration efficiency. Subsequent efforts incorporated prior knowledge about amino acid physicochemical properties, achieving notable successes by introducing rudimentary priors related to plausibility [12, 13].

Existing computational methods for protein evolution, such as SIFT [14], PROVEAN [15], PolyPhen-2 [16], and PANTHER [17], essentially address a common problem: evaluating the “plausibility” of mutations relative to evolutionary history. The underlying rationale posits that sequences conserved during evolution are plausible, where sequence conservation was measured by different approaches. Specifically, these methods can be summarized into the following three categories based on the type of prior information they integrate: Methods based on multiple sequence alignments (e.g. SIFT’s conservation score [14], PANTHER’s evolutionary model [17], or DeepSequence’s Variational Autoencoder [18]) capture conservation and co-evolution patterns in natural evolution. Methods based on physicochemical property matrices (e.g. Grantham [19] and BLOSUM62 [20]) are interpretable physical–chemical basis measures. Methods based on structural features (e.g. SuSPect [21], NetDiseaseSNP [22]) assess the potential impact of mutations on structural stability. Although employing distinct technical approaches, all these methods ultimately aim to integrate the prior knowledge of “historical evolutionary plausibility” into their algorithms.

**Figure 1:**
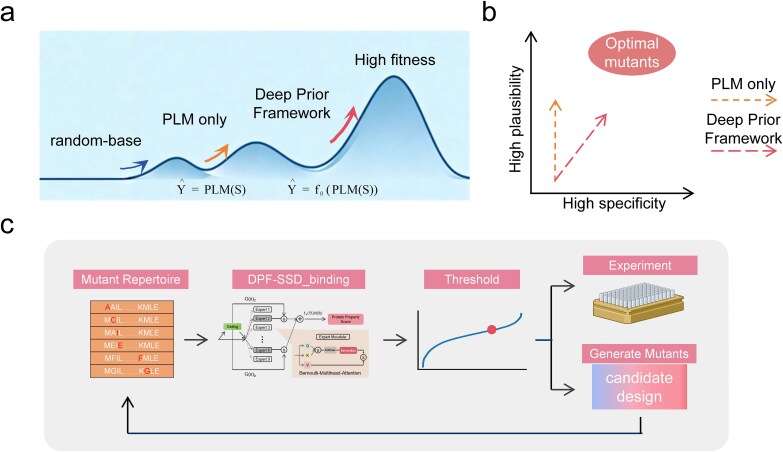
Guiding protein evolution with prior information. (a) The objective of protein evolution is to identify high-fitness mutants. Using statistical optimization as an analogy, the ultimate goal is to find the global optimum, while the strategy (or model) employed determines the local optimum attainable. More accurate prior information can significantly enhance the efficiency of discovering high-fitness mutants. Compared to random-based methods such as error-prone PCR (left), approaches that leverage PLM logits to infer amino acid substitution probabilities provide substantially richer prior evolutionary information (Middle). For personalized evolutionary objectives, we further propose that introducing additional targeted prior information (right) could lead to even greater efficiency. (b) PLM-only methods utilize the common evolutionary plausibility of proteins as prior information. However, for specific protein engineering tasks, there exist additional, more personalized requirements. The multiple classification datasets used to train the DPF incorporate diverse evolutionary information for various properties. We hypothesize that DPF can learn a portion of this personalized prior knowledge. If this hypothesis holds true, DPF would possess a more direct capability than PLM-only methods to identify optimal mutants. (c) The flowchart illustrates the main workflow and application scenarios of DPF. After obtaining the embeddings of mutants using a PLM, DPF can provide predictive scores. These scores can be used either to screen mutants for the first round of experimental testing or to directly generate combinatorial mutant libraries for iterative protein evolution *in silico*.

Advances in computational science have enabled the emergence of large-scale Protein Language Models (PLMs), such as the widely applied ESM2 [23], has catalyzed a shift toward deep-learning-based representations of evolutionary plausibility. By quantifying the likelihood of different mutants through logit layers, these models learned representation of evolutionary plausibility [23–33]. This capability stems from PLMs learning a substantially enriched body of protein prior information from extensive protein sequence databases. Building on this, researchers have established zero-shot PLM-based approaches (Fig. 1a, middle), which achieved remarkable results in engineering proteins, notably antibodies [6, 34, 35] based on only two–five rounds of experimental validation to yield significantly improved mutants.

However, without experimental feedback, the top-ranked mutants identified by these methods often show minimal or even slightly negative performance [6, 34–37]. In addition, current PLMs are primarily trained on known sequences from databases like UniProtKB, which represents only a fraction of the vast plausible protein space [38, 39]. Meanwhile, human objectives for protein engineering are highly diverse. Although all proteins share a common core of evolutionary plausibility [6], there exists a wide spectrum of specific, tailored evolutionary demands. This raises a critical question: Can we develop more advanced methodologies that not only incorporate general plausibility but also efficiently guide protein evolution according to “specific functional requirements”? In other words, can we provide more targeted prior information about the desired evolutionary trajectory (Fig. 1a, right)?

To address this challenge, we propose the Deep Prior Framework (DPF), a framework utilizes the embedding layers of PLMs to encode protein sequences [28]. It incorporates prior knowledge for directed protein evolution by pretraining a model on external categorical labels related to specific protein functions or properties (Fig. 1b, c). This framework not only closes integration with experiments—allowing the first round of screening to enrich more high-fitness mutants—but also facilitates purely computational evolution of target proteins, significantly reducing experimental costs (Fig. 1c). Our results demonstrate that pretrained models incorporating such prior information can significantly enhance the efficiency of protein evolution. We validated the reliability and effectiveness of the DPF method across nine Deep Mutational Scanning (DMS) datasets. Finally, we present a case study involving the *in silico* evolution of *Blastobotrys adeninivorans* xanthine dehydrogenase (*Ba*XD), with experimental results confirming the strong performance of DPF in a practical scenario.

## Results

### The architecture of deep prior framework and model performance

Here we develop a deep learning-based framework for directed protein evolution, termed the DPF. DPF comprises two key components: (i) a PLM that embeds protein sequences into a latent space to obtain their high-dimensional representations, thereby providing prior information on evolutionary “plausibility”; (ii) a classification model based on a Mixture of Experts (MoE) architecture [40, 41]. The classification model incorporates a Bernoulli-Attention (BATT) module as its “expert” to provide prior information on task-specific “specificity”. Unlike directly using the model’s logits to score the plausibility of amino acid choices at each position, the embeddings reside in a higher-dimensional space and are presumed to encapsulate more comprehensive sequence evolutionary features, making them more suitable for our subsequent classification task. While MoE architecture helps our model remain computationally lightweight while learning to discriminate between positive and negative sequences from diverse perspectives while BATT “expert” not only potentially accelerates model inference but also enhances model accuracy by encouraging sharper attention distributions.

We selected nine commonly studied protein properties and defined representative positive and negative protein subsets for each property. These subsets were used to train nine independent DPF classification models. This design aims to guide the models to learn the principal distinctions between positive and negative examples. To ensure the learning objective aligns with our intended direction and to mitigate potential directional biases, we maximized the diversity of protein types within each positive/negative set. For instance, the negative set for single-strand DNA-binding (SSD-binding) proteins included five distinct protein categories: dCMP deaminase proteins, oxidoreductase proteins, secreted proteins, transferases proteins, and transmembrane proteins (Supplementary Table S1, Supplementary data S1).

To evaluate the rationale behind our model design, we compared three architectural variants across the nine classification datasets under 40 consecutive random seeds each:

(1) A model using solely standard Attention (ATT) layers.
(2) A model using the MoE architecture, where each expert employs standard ATT modules (MoE-ATT).
(3) A model using the MoE architecture, where each expert employs our proposed B-ATT modules (MoE-BATT).

**Figure 2:**
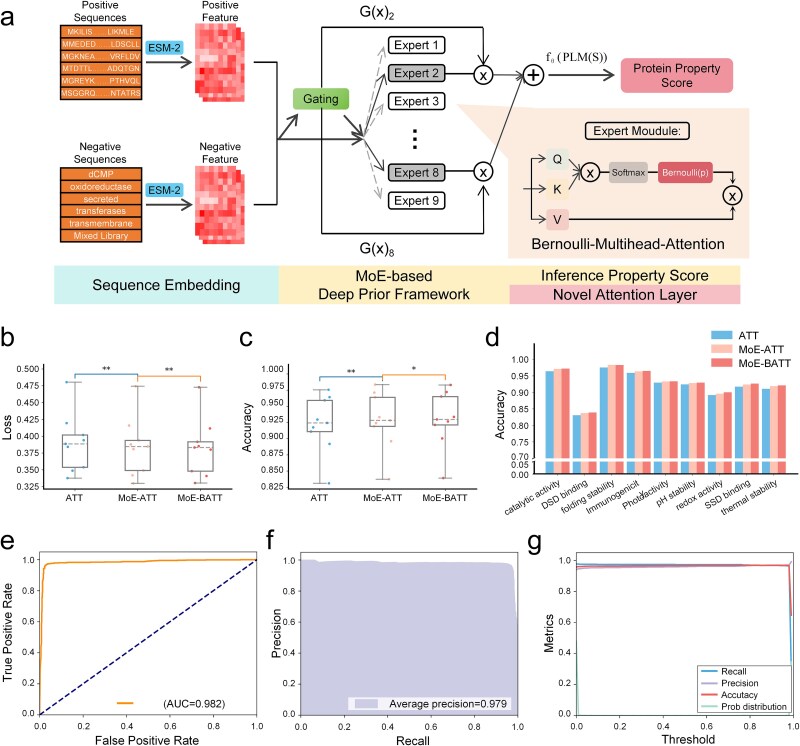
High-accuracy DPF with the BATT Module. (a) Flowchart of the DPF. The training set for each DPF consists of positive and negative examples. Protein sequences are embedded using ESM-2 (esm2_t33_650M_UR50D), and the resulting embedding matrix serves as the model input. The core of the model is a MoE-based binary classifier. A gating network dynamically weights the contributions of nine “Experts”, each comprising our proposed BATT module. A key innovation in this module is performing a Bernoulli sampling step for each element in the attention matrix. The final output of the DPF is a score for a given protein sequence. (b–d) We trained nine distinct DPF models, each for a different property. Model performance was evaluated on the test set using the average results from 40 consecutive random seeds. Three different model architectures were compared. Box plots display the accuracy (and loss) performance of different model architectures across the nine datasets (b, c). The MoE-BATT architecture achieved a significantly lower loss (b) and a significantly higher accuracy (c) than the alternatives, a Wilcoxon Signed-Rank Test was used to calculate the *P*-value between each two groups (*P < .05, **P < .01). The specific performance for each of the nine DPF models is shown as a Bar plot (d). (e–g) Detailed binary classification performance of the DPF-SSD_binding model on the test set, including the ROC curve (e), the Precision-Recall curve (f), and the distribution of Recall, Precision, and Accuracy across different classification thresholds, alongside the distribution of prediction scores on the test set (g).

Benchmarking on the above datasets, the MoE-ATT architecture demonstrated statistically significant superiority over the ATT-only architecture in terms of loss and accuracy (Wilcoxon Signed-Rank Test, *P* < .05), confirming the effectiveness of incorporating the MoE framework. Furthermore, the MoE-BATT architecture significantly outperformed the MoE-ATT architecture (Wilcoxon Signed-Rank Test, *P* < .05) (Fig. 2b and c). For the classification task corresponding to each individual protein property, the MoE-BATT model consistently achieved the best performance (Fig. 2d). Taking the model for the SSD-binding property as an example, it achieved an ROC-AUC of 0.982 and an Average Precision of 0.979 on the test set (Fig. 2e and f). Moreover, the classification scores were strongly concentrated near 0 and 1 (Fig. 2g), suggesting high confidence in predictions and providing a wide threshold selection range for practical applications. The classification metrics for models corresponding to the other properties showed similarly strong performance (Table 1, Supplementary Figs S1–S4, Supplementary Table S2).

**Table 1:** Performance of the nine DPF models on the test set.

	loss	acc	precision	recall	auc	ave_precision
Catalytic activity	0.416	0.895	0.947	0.837	0.947	0.946
DSD binding	0.340	0.974	0.980	0.967	0.991	0.988
Folding stability	0.391	0.923	0.937	0.906	0.950	0.942
Immunogenicity	0.487	0.823	0.822	0.825	0.889	0.867
Photoactivity	0.348	0.965	0.965	0.965	0.978	0.966
pH stability	0.427	0.884	0.913	0.848	0.928	0.931
Redox activity	0.391	0.922	0.931	0.911	0.954	0.959
SSD binding	0.346	0.966	0.960	0.973	0.982	0.979
Thermal stability	0.397	0.913	0.946	0.875	0.949	0.960

### Efficient enrichment of positive mutations across diverse protein families

To evaluate the reliability and generalizability of DPF for protein engineering, we assessed its predictions on multiple DMS datasets [42–51]. These datasets provide experimental gold-standard measurements alongside predictions from dozens of established methods, enabling a fair, standardized comparison of DPF against other models.

**Figure 3:**
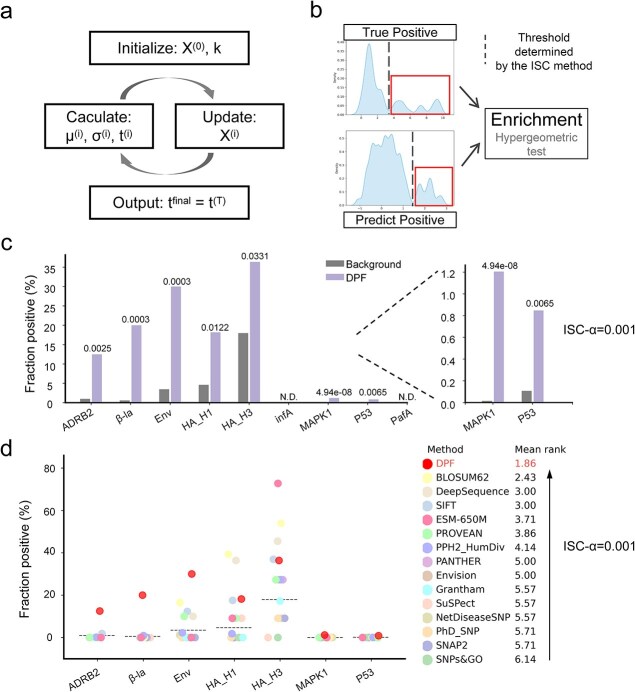
Benchmarking DPF on Multiple Protein Families. (a) Schematic diagram of the ISC algorithm (see Methods for details). (b) For each DMS dataset and its corresponding predictions from various methods, the ISC algorithm was applied to determine positive thresholds, enabling subsequent comparative analysis. (c) Bar plots show the proportion of positives in the DMS dataset (grey) and the proportion of positives identified by the DPF score (purple), after applying the ISC-defined positive threshold at α = 0.001. For InfA and PafA at α = 0.001, the experimental values fell below the calculated positive threshold (N.D.). Results for MAPK1 and P53 are shown on a different scale (right). A one-sided hypergeometric test was used to calculate the *P*-value for enrichment in each protein family. (d) For each protein family, the same number of top-scoring mutants as selected by DPF was chosen from the predictions of every other method to define their respective recommended sets (see Methods). The proportion of true DMS positives within each method’s selected set is compared across all methods (left). Methods were then ranked based on this proportion within each family, and the average rank across all families was computed for each method (right).

Prior research has established that language model-based predictions correlate well with experimental fitness measurements across libraries ranging from ~10^1 to 10^3–10^4 variants [6, 52, 53]. Since DMS datasets lack a universally defined threshold for classifying “positive” mutations, and the quantities used in prior studies for rank filtering span multiple orders of magnitude, we designed a data-driven method for positive set delineation called Iterative Sigma-Clipping (ISC). This iterative algorithm dynamically determines a positivity threshold based on the underlying distribution of the experimental data (Fig. 3a; Supplementary Fig. S8; Methods). We applied ISC independently to define positive mutation sets for the DMS gold standard, DPF predictions, and all other model predictions. The comparison between any model’s recommended set and the gold-standard positive set then becomes an enrichment problem, evaluated using a one-sided hypergeometric test (Fig. 3b).

DPF-recommended beneficial mutants showed significant enrichment (one-sided hypergeometric test, *P* < .05) in all seven datasets where a positive set was identifiable by ISC (for infA and PafA datasets, no mutations surpassed the ISC-defined threshold at α = 0.001). In each of these seven datasets, the proportion of true positives within the DPF-recommended subset was substantially higher than the background proportion in the full mutant library (Fig. 3c, Supplementary Fig. S7). For instance:

- In the β-lactamase (β-la) single-point mutant library, the background positive mutation rate was <0.58%. In contrast, the rate within the DPF-recommended set reached 20%, representing a 34.6-fold enrichment over background.
- In the Envelope (Env) protein mutant library, the background rate was below 3.48%, while the rate within the DPF recommendations was 30%, corresponding to an 8.6-fold enrichment.

We computed the enrichment performance for dozens of alternative methods provided with the DMS benchmarks—including supervised and structure-based models—and additionally included the method from Hie et al. [6]. We ranked all methods for each dataset based on the proportion of true positives within their recommended mutant sets. Considering the average rank across all seven informative datasets (α = 0.001), DPF achieved the highest overall rank. Notably, DPF attained the top rank in five out of the seven datasets (Fig. 3d, Supplementary Fig. S9), and also consistently outperformed the PLM-based method (ESM-650M) across six datasets. To ensure that the average-rank metric did not obscure meaningful differences in effect size, we additionally computed three aggregate performance metrics averaged across the informative datasets: (i) the mean of 1—P-value, (ii) the mean of -log₁₀(P-value), and (iii) the mean fraction of true positive mutations recovered. The results demonstrate that DPF consistently achieves the best performance on all three of these aggregate metrics (Supplementary Fig. S10), further confirming its superior capability to enrich for beneficial mutations.

Building on the observed performance differences between DPF and the PLM-based method, we further examined the relationship between ESM plausibility scores and our model's specificity scores across the DMS datasets (Supplementary Fig. S11). The scatter plots reveal that the data points are broadly distributed without a discernible trend or pattern. Consistently, the Pearson correlation coefficients between the two scores are all close to zero (0.0045 ± 0.0117), indicating that they capture nearly orthogonal patterns of sequence variation. This supports the notion that plausibility and specificity represent distinct, largely independent dimensions of the fitness landscape, which may explain why DPF's specificity-driven enrichment strategy achieves superior performance over PLM-based approaches.

### *In silico* guided efficient evolution of the *Blastobotrys adeninivorans* xanthine dehydrogenase molybdenum-pterin domain

Current methods typically require two–five rounds of experimental feedback to obtain significantly improved protein variants [6, 35]. However, we observed that the initial performance gains from the top mutants recommended in the very first round by such models are often minimal or even slightly negative [6, 34–37]. Therefore, we sought to demonstrate that efficient protein evolution could be achieved relying solely on *in silico* guidance, without intermediate experimental data to “correct” the evolutionary trajectory.

We selected the molybdenum-pterin (MOCO) domain—one of the primary structural domains of BaXD—as the target for our evolution. BaXD catalyzes the oxidation of xanthine to uric acid, a reaction mechanism chemically analogous to the oxidation of guanine to 8-oxoguanine (8-oxoG), suggesting its potential as a base transversion editing tool. The core structural domains of BaXD include a MOCO domain, two iron–sulfur clusters, and a flavin adenine dinucleotide cofactor. The molybdenum ion, coordinated within the MOCO cofactor, constitutes the enzyme’s catalytic center, while the other two domains primarily facilitate electron transfer. However, the inability of xanthine oxidase to act directly on single-stranded DNA significantly limits its editing efficiency as a base editor. To address this limitation, we aimed to enhance its DNA-binding capability to improve editing performance.

We first applied the DPF-SSD_binding model to score the wild-type *Ba*XD MOCO domain and all its single-point amino acid mutants. The model predicted that their SSD-binding capability was exceedingly low (score < 1 × 10^−15^, Supplementary Fig. S12a). This result, on one hand, supported our hypothesis that enhancing SSD-binding is a critical and required evolutionary direction for this protein. On the other hand, the uniformly low scores for all single mutants indicated the potential difficulty of this engineering task.

**Figure 4:**
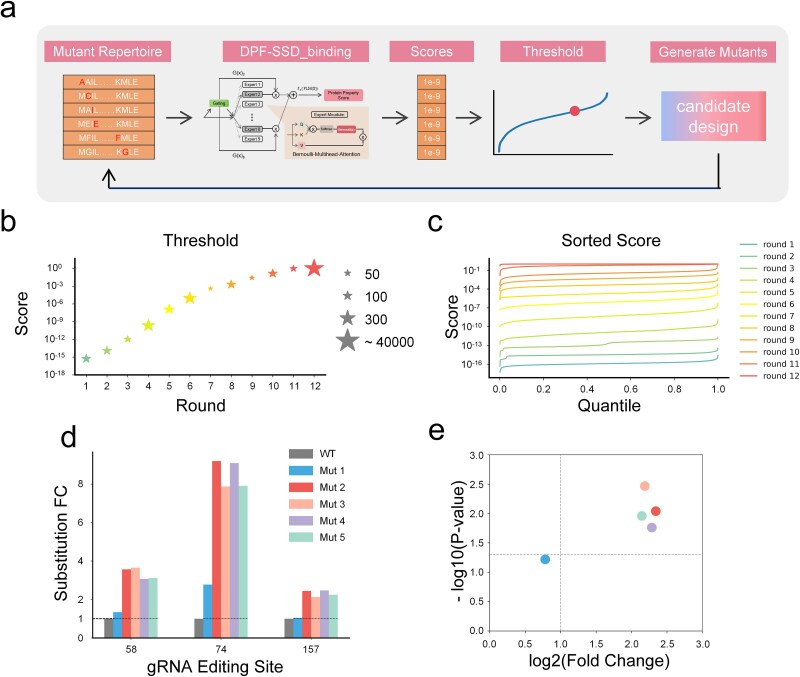
DPF-guided enhancement of enzymatic activity of the BaXD MOCO Domain. (a) Workflow of the in silico protein evolution. Starting from an initial candidate mutant repertoire, variants are scored by DPF-SSD_binding. The ranked scores are analyzed using the ADF test to determine a threshold for selecting mutants to constitute the repertoire for the next round (see Methods). (b) Scatter plot showing the threshold determined for each round (log-scale y-axis). The size of each point corresponds to the number of mutants exceeding the threshold in that round. In the final round (Round 12), scores were uniformly high, so the threshold was empirically set to 0.95, resulting in approximately 40 000 mutants exceeding it. (c) Line plot illustrating the change in scores of the candidate mutant repertoires across the 12 rounds. (d) Bar plot visualizing the base editing frequency at three gRNA editing sites for the wild-type (grey) and the five selected mutants. (e) Scatter plot displaying the log2 fold-change in enzymatic activity relative to wild-type (x-axis, mean calculated across the three sites) versus the associated -log10(p-value) (y-axis, *P*-value from a two-sided paired *t-*test across the three sites) for each mutant.

Consequently, we adopted a straightforward strategy: iteratively combining and extending mutations to ultimately identify mutants that the DPF-SSD_binding model would score close to 1, followed by experimental validation. Specifically, in each round, we scored the candidate mutant library using the model, ranked the scores, and applied the Augmented Dickey–Fuller (ADF) test to identify variants scoring above a dynamically set threshold. Promising mutants from this round were then used as templates to design a subsequent candidate library incorporating further mutations via combinatorial exploration. This iterative process was repeated until mutants receiving high model scores were obtained (Fig. 4a; Methods).

We conducted 12 rounds of *in silico* evolution. Both the selection threshold and the average model score of the candidate pool increased progressively with each round (Fig. 4b and c; for detailed, see Supplementary data S2 and Supplementary Fig. S12). Finally, we selected the top five highest-scoring mutants for experimental characterization. Compared to the WT, these five mutants exhibited an average increase in activity exceeding four-fold across three tested sites. The best-performing mutant demonstrated over a nine-fold enhancement in activity relative to WT (Fig. 4d and e).

### Mutation preference in protein evolution and its validation

In previous section, we observed that a substantial proportion of the mutated sites in the five final selected variants were substituted with L-Cysteine (Cys). To investigate whether this preference was stochastic or a confident prediction by the model, we conducted a series of systematic replacement experiments using the high-performing Mut2 as a template. It is important to note that in all these experiments, the original non-cysteine mutations present in Mut2 were retained.

First, we generated new mutants by selecting 1 to 15 positions from the set of all cysteine mutations found in Mut2. For combinations where the total number exceeded 1000, we randomly sampled 1000 instances. The results demonstrated a strong, approximately log-linear increase in the model-predicted score with the number of introduced cysteine mutations, with scores plateauing near the maximum value of 1 (Supplementary Fig. S13a). Next, we individually mutated each wild-type (non-cysteine) position not already altered in Mut2 to cysteine. The model scores for mutants incorporating the specific cysteine substitutions present in Mut2 were significantly higher (unpaired two-tailed *t-*test *P*-value = 3.18 × 10^−11^) than those for mutants involving other cysteine substitutions (Supplementary Fig. S13b). Finally, we performed 10 000 random simulations, in each case selecting 15 random wild-type (non-cysteine) positions to mutate to cysteine. The analysis revealed a clear positive correlation: the more a random set of 15 cysteine mutations overlapped with the specific set found in Mut2, the higher the model score assigned to the resulting mutant (Supplementary Fig. S13c).

Collectively, these results provide strong computational evidence that the prevalence of cysteine mutations at specific positions in the top variants is not random. Instead, the model confidently identifies these specific cysteine substitutions as being highly beneficial for enhancing the protein’s activity.

### Deep prior framework predicts high-confidence labels for the UniProt unreviewed dataset

The UniProtKB [54] currently contains 199 006 240 unreviewed entries, which is approximately 347 times larger than reviewed proteins. This stark disparity underscores the growing importance and urgency of automated protein annotation [28, 55]. Given the exceptional performance of our models on validation and test sets, these nine DPF classifiers are highly suitable for large-scale prediction across the entire unreviewed protein database. By applying a very high score threshold (0.999), we assigned high-confidence labels to these proteins. This approach establishes a valuable high-confidence label dataset, which holds significant potential both for guiding curators toward proteins most likely to possess specific functions for experimental verification and for serving as enriched training data to develop even more powerful next-generation models.

**Figure 5:**
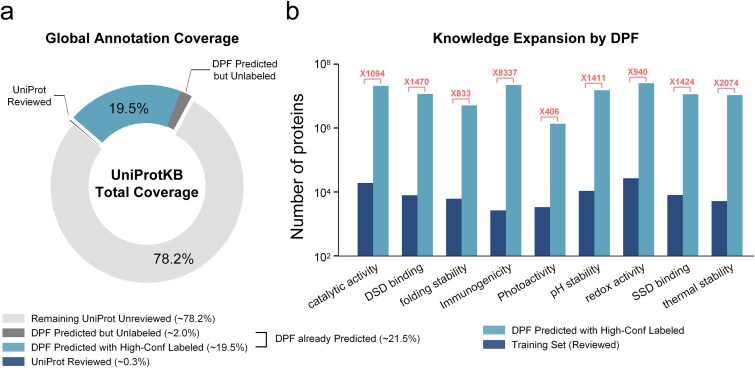
Providing predicted labels for the UniProt unreviewed dataset. (a) Pie chart showing the proportion of reviewed proteins in UniProtKB (dark blue). Within the unreviewed section, the chart shows the proportion for which we have generated DPF predictions and obtained at least one high-confidence label (light blue), the proportion that did not receive any label (dark gray), and the proportion of unreviewed proteins not yet predicted (light gray). (b) Bar graph displaying the number of positive examples (from UniProt reviewed) in the training set (dark blue) for each of the nine DPF models, compared to the number of high-confidence labels assigned within the predicted portion of the UniProt unreviewed dataset (light blue).

Based on the above procedure, we have generated predictions for 42 913 366 unreviewed proteins (~21.56% of the total). Using an exceptionally high confidence threshold (0.999)—which corresponds to an average precision of 95.58% on the test set (Supplementary Fig. S3, Supplementary data S3)—we assigned model-derived labels. Even under a very stringent threshold, 90.07% (38 645 581) of the predicted proteins can receive at least one label (Fig. 5a). Categorized by the nine DPF models, this yields an average of approximately 13 845 625 high-confidence labels per property (including 11 545 549 for SSD-binding), representing an average 1999-fold increase in annotated examples compared to the manually reviewed positive sets in UniProt (Fig. 5b). This massively expands the universe of proteins with functional annotations. Incorporating this annotated data into a semi-supervised learning framework can substantially expand the effective dataset size and enhance model performance, while also providing a reliable preliminary screening resource for subsequent experimental validation.

### Validation on the UniProt TrEMBL dataset

To rigorously assess model generalization, we validated our framework on an independent dataset derived from the unreviewed TrEMBL section of UniProt. In detail, based on the same training pipeline, we transitioned the data source from the manually curated Swiss-Prot database to the automatically annotated TrEMBL database. We extracted sequences from TrEMBL labeled as positive or negative based on the same functional definitions used for training. The resulting validation set contained 9 704 500 unique sequences labeled as positive.

For positive TrEMBL sequences, our model achieved a mean prediction score of 0.909. Notably, 91.0% of sequences scored above 0.5, and 85.3% scored above the stringent 0.999 threshold. These results indicate that the model can identify the vast majority of automatically curated positive examples with high confidence. Conversely, for TrEMBL sequences labeled as negative, the model’s mean prediction score was only 0.082, with merely 8.0% scoring above 0.5 and only 3.5% exceeding 0.999. This performance demonstrates the model’s robust discriminative power and low false-positive rate on unseen, lower-quality annotated data. Overall, this validation confirms that our model can robustly and confidently identify sequences that conform to functional definitions, even when applied to datasets with varying annotation quality and provenance.

## Discussion

We validated the exceptional performance of DPF on DMS datasets, where it achieved the highest average ranking, and successfully enhanced the activity of the *Ba*XD MOCO Domain by an average of four-fold through a purely computational approach. Our findings demonstrate that pre-trained models incorporating targeted prior information can effectively guide protein engineering tasks. Specifically, we show that models based on the same foundational architecture (ESM2) achieve superior performance when augmented with functional priors (Fig. 3d). This strategy is particularly advantageous when evolving proteins toward well-defined functional properties.

From a broader perspective, the vastness of possible protein sequence space remains immense, rendering exhaustive exploration infeasible even with foreseeable advances in computational power. This fundamental constraint necessitates the integration of richer prior information to navigate the fitness landscape efficiently. Only by strategically incorporating prior information to reduce the uncertainty inherent in this vast search space can we hope to identify functional peaks—even those residing in low-probability regions—with practical efficiency.

In essence, the history of protein engineering reflects a continuous effort to incorporate more prior knowledge. The field has progressed from leveraging simple physicochemical amino acid properties, to utilizing statistical patterns from entire protein families via PLMs, and now to our approach of integrating explicit functional objectives through DPF models. Each step represents a paradigm of supplying more informative constraints, thereby systematically reducing the search space and accelerating the discovery of proteins with desired functions.

PLMs will become an indispensable foundation for protein design and evolution. Compared to traditional high-throughput experimental screening strategies, PLMs offer unparalleled advantages, including robust zero-shot prediction capabilities and the computational efficiency of *in silico* evaluation, making them powerful tools for researchers. As our understanding of proteins deepens, the objectives of protein engineering continue to evolve, with increasingly specific and elaborate demands emerging. Relying solely on the generic evolutionary plausibility captured by standard PLMs may be insufficient to meet these highly tailored requirements. Our DPF addresses this by incorporating specific functional objectives, enabling more controlled and efficient protein evolution toward predefined goals, as demonstrated with the nine distinct DPF models developed herein.

A key feature of the DPF approach is its reliance on learning the distinctions between user-defined positive and negative protein datasets. This means that for any new functional target, generating a specialized DPF primarily requires curating relevant protein sets—a task feasible using existing databases like UniProtKB, without the immediate need for large-scale experimental data. Thus, the significance of this work extends beyond the specific properties explored here; it provides a low-cost, reproducible, and generalizable modeling framework for the community.

It is important to note that this study was conducted using the relatively small ESM2-650M model due to computational constraints. Empirical evidence suggests that utilizing larger PLMs (e.g. ESM2-3B or 15B) for generating sequence embeddings can objectively enhance model performance [35]. Furthermore, the framework is inherently adaptable to future advances in PLMs. For specialized tasks such as antibody engineering, one could readily substitute a dedicated antibody language model for sequence embedding. The framework could even be extended to integrate embeddings from multiple PLMs, allowing the model to learn from diverse protein representations. Given sufficient computational resources, exploring such multi-model integration represents a promising direction for future work.

Our model demonstrated robust performance across DMS benchmarks at various significance levels defined by the ISC method (Supplementary Figs S7, S9, Supplementary Table S3), with peak performance observed at the most stringent threshold (α = 0.001). This indicates a heightened sensitivity for identifying the most statistically significant beneficial mutants. For practical protein engineering campaigns, this characteristic is advantageous as it suggests that screening a smaller, top-confidence candidate set recommended by DPF is likely to capture the highest-potential variants, thereby improving experimental efficiency.

Further validating the quality of DPF’s predictions, at the α = 0.001 threshold, the top-scoring mutant identified by DPF in five of the seven informative DMS datasets exceeded the 99th percentile of the experimental fitness distribution. The top candidates in the remaining two datasets also approached this high benchmark closely (Supplementary Table S3).

The *in silico* evolution of the *Ba*XD MOCO domain using DPF-SSD_binding, while successful, involved several heuristic steps. Retrospective analysis of this process yields valuable insights for optimizing future workflows. For instance, the strategy of performing pan-single-mutation scans on top-performing candidates was only introduced after round seven. However, analysis of mutation site preferences suggests that implementing this strategy as early as round three (Supplementary Fig. S14) could have helped escape local optima more efficiently by exploring a broader mutational landscape beyond combinations of initially fixed mutations.

The observed enrichment of cysteine (Cys) substitutions in the optimized BaXD MOCO domain warrants further mechanistic scrutiny. First, to rule out a trivial bias in the model, we quantified the frequency of Cys residues in the training data. In the positive (high-activity) and negative (low-activity) sequence sets used for training the specificity model, Cys frequencies were 1.38% and 1.68%, respectively. Both are substantially lower than the random expectation of 5% (1/20), and the frequency is even slightly lower in the positive set. This indicates that the model did not simply learn to increase Cys abundance from the data distribution; rather, it had to overcome an inherent data bias “against” Cys to predict its benefit. Consistent with a position-specific rationale, the model assigned significantly higher scores to Cys substitutions at the predicted sites compared to other amino-acid substitutions at the same positions or to Cys substitutions at random positions in the protein (Supplementary Fig. S13), confirming that the preference is site-specific, not a general propensity.

A particularly intriguing observation emerged from comparing the highly improved mutants (mut2–5) to the modestly performing mut1. Structural analysis (Supplementary Fig. S15) revealed that those six mutations that only appear in mut2–5 and are different from mut1 were located on the protein surface, away from the canonical active site pocket. This suggests that the model identified beneficial “key mutations” in regions traditionally considered less critical for function. This finding aligns with growing evidence that long-range, allosteric mutations distant from the active site can significantly enhance protein activity [56–58], highlighting the model’s ability to uncover non-obvious design rules beyond human intuition.

## Method

### Data embedding

For all protein sequences, we employed PLM to generate their initial embeddings. Specifically, for each protein sequence of length $L$, we used a PLM to produce an initial representation in the form of an $L\times d$ embedding matrix (this study utilized the esm2_t33_650M_UR50D, where *d* = 1280). Each row in this matrix corresponds to the $d- dimensional$ contextual embedding vector of an amino acid residue.

To convert variable-length protein sequences into fixed-dimensional input features, we applied a global average pooling operation. Specifically, we performed average pooling along the sequence length dimension (the first dimension) of the ESM2 output matrix:


$$ \boldsymbol{x}=\frac{1}{L}\ {\sum}_{i=1}^L\ {h}_i $$

where ${h}_i\in{R}^d$ represents the embedding vector of the $i- th$ residue in the sequence. This operation yields a global feature vector $\boldsymbol{x}\in{R}^d$ for a protein. This vector aggregates contextual information from the entire sequence and serves as the standard input for our model.

### Bernoulli-Attention module

We innovatively designed the BATT module as an enhancement to the standard multi-head self-attention mechanism [59], aiming to mitigate overfitting and improve generalization by introducing structured stochasticity.

Given an input representation matrix $\boldsymbol{x}$ for a sequence, we first compute the Query (*Q*), Key (*K*), and Value (*V*) matrices identically to the standard mechanism:


$$ Q=\boldsymbol{x}{W}^Q,K=\boldsymbol{x}{W}^K,V=\boldsymbol{x}{W}^V $$

where ${W}^Q,{W}^K,{W}^V$ are learnable weight matrices. Subsequently, $Q$, $K$, and $V$ are split into multiple heads. The following process is applied identically within each head.

The scaled dot-product attention score matrix $A$ is computed as:


$$ A=\mathrm{Softmax}\left(\frac{Q{K}^T}{\sqrt{d_k}}\right) $$

where is the dimensionality of the key vector $K$. The scaling factor $\frac{1}{\sqrt{d_k}}$ is used to prevent the dot product results from having excessively large variance when ${d}_k$ is large, which would cause the $Softmax$ function to enter saturation regions with minimal gradients, thereby affecting model training.

We introduce a Bernoulli sampling layer after the $Softmax$ operation. Specifically, for each element ${A}_{ij}$ in the attention matrix $A$ (representing the attention weight from the $i- th$ token to the $j- th$ token), we perform Bernoulli sampling using its probability value, generating a binarized attention mask matrix $M$:


$$ {M}_{ij}\sim \mathrm{Bernoulli}\left({A}_{ij}\right) $$

The Bernoulli sampling operation is inherently non-differentiable. To enable end-to-end training, we employ the straight-through estimator for gradient approximation. During the forward pass, the discrete Bernoulli sample is used as described. In the backward pass, however, the gradient with respect to the sampling probability is approximated by directly passing the gradient through the discrete sampling step as if the thresholding operation were the identity function. Formally, the gradient of the loss with respect to the input probability is approximated as:


$$ \frac{\partial \mathcal{L}}{\partial{A}_{ij}}\approx \frac{\partial \mathcal{L}}{\partial{M}_{ij}} $$

The final output is obtained by multiplying the sampled binarized mask $M$ with the value matrix $V$. Since this is multi-head attention, the results computed separately for each head need to be concatenated:


$$ E=\mathrm{concat}\left({M}_1{V}_1,\dots, {M}_h{V}_h\right) $$

where $h$ represents the number of heads in the model.

### Model architecture of the deep prior framework

DPF is a deep learning model based on the MoE architecture. We introduced the MoE framework to efficiently scale the model’s capacity through conditional computation. Unlike traditional feed-forward neural networks, an MoE layer consists of multiple “Expert” networks. For each input, a trainable gating network dynamically selects the most relevant subset of experts for processing, while the remaining experts remain inactive.

In our implementation, each expert ${E}_k$ is an independent Bernoulli-Attention module, specifically:


$$ {\mathrm{E}}_{\mathrm{k}}\left(\boldsymbol{x}\right)=\mathrm{BATT}\left(\boldsymbol{x}\right),\mathrm{for}\;\mathrm{k}=1,\dots, \mathrm{N} $$

where $\boldsymbol{x}$ is the embedding representation matrix of the protein sequence, $k$ represents the $k- th$ expert, and $N$ is the total number of experts in the MoE.

The gating network $G\left(\boldsymbol{x}\right)$ is responsible for generating a probability distribution over the $N$ experts based on the input $\boldsymbol{x}$. Its structure is a simple two-layer feed-forward neural network, containing one hidden layer with a $ReLU$ activation function.

First, the input vector $x$ is projected into an $h$-dimensional hidden space:


$$ h=\mathrm{ReLU}\left(x{W}_{\mathbf{1}}^{\top }+{b}_{\mathbf{1}}\right) $$

where ${W}_1\in{R}^{h\times d}$, ${b}_i\in{R}^h$.

Subsequently, the hidden representation $h$ is transformed into $N- dimensional$ raw gating logits:


$$ {g}^{\prime }=h{W}_2^{\top }+{b}_2 $$

where ${W}_{\mathbf{2}}\in{R}^{N\times h}$, ${b}_{\mathbf{2}}\in{R}^N$.

Finally, the normalized gating weights are obtained by applying the Softmax function to the logits:


\begin{align*} G(x)&=\mathrm{Softmax}\left({g}^{\prime}\right)\\&=\left[\frac{\exp \left({g}_1^{\prime}\right)}{\sum_{j=1}^N\exp \left({g}_j^{\prime}\right)},\frac{\exp \left({g}_2^{\prime}\right)}{\sum_{j=1}^N\exp \left({g}_j^{\prime}\right)},\dots, \frac{\exp \left({g}_N^{\prime}\right)}{\sum_{j=1}^N\exp \left({g}_j^{\prime}\right)}\right] \end{align*}

Therefore, the final output of the MoE layer is the weighted sum of the outputs from the selected experts. Let $Top(n)- Indices$ be the set of indices corresponding to the $n$ experts with the highest gating weights. The output $y$ is then given by:


$$ y={\sum}_{i\in \mathrm{TopKIndices}}G{\left(\boldsymbol{x}\right)}_i\cdotp{E}_i\left(\boldsymbol{x}\right) $$

where $G{\left(\boldsymbol{x}\right)}_k$ represents the gating weight for the $k- th$ expert given the input $\boldsymbol{x}$.

### Deep prior framework model training

We began by merging the nine datasets and utilized Python’s Optuna library to perform Bayesian optimization of hyperparameters (learning rate, weight decay, and number of experts) under a fixed random seed of 42. Subsequently, we trained nine separate DPF models, each corresponding to one of the protein properties, while maintaining the same random seed (42). The model architecture adopts an MoE structure, with an input dimension of 1280 (corresponding to ESM2 embeddings). It consists of nine expert networks and a gating network with a hidden dimension of 256. The CrossEntropyLoss was employed as the loss function, and the AdamW optimizer (β₁ = 0.9, β₂ = 0.999) was used with a base learning rate of 1.06e-4 and weight decay of 1.06e-6. Training was performed with a batch size of 32 over 30 epochs. Validation was conducted after every epoch, and model checkpoints were saved every five epochs. The data for each property were split into training, validation, and test sets in an 80%: 10%: 10% ratio. During the 30 training epochs, the model with the highest validation accuracy was selected and its performance was evaluated on the test set.

For the comparative analysis of the three different model architectures (ATT, MoE-ATT, MoE-BATT), we employed 40 consecutive random seeds (10 to 49) to assess model performance robustly. All other training parameters and procedures remained consistent with the description above.

### Computational resources and costs

To ensure full transparency and reproducibility, we report the computational resources required for training and inference.

#### Training cost

Training of a single property-specific DPF model for 30 epochs (with a batch size of 32) was conducted on a single NVIDIA V100 GPU (32GB memory). The total training time per model was approximately 4 minutes.

#### Inference cost

The inference time was evaluated on a typical DMS dataset containing ~10 000 mutations. Using the same GPU (NVIDIA V100) with a batch size of 128, the inference process for the DPF model averaged 20 s.

#### Important note on preprocessing

The inference time reported above does not include the preprocessing step of converting protein sequences into ESM-2 (650M parameters) embeddings. This embedding generation, which relies on the upstream ESM model, is a one-time, cacheable fixed cost. Generating embeddings for 10 000 sequences (with a batch size of 1) required approximately 130 min on the same hardware. This distinction is made to provide a clear assessment of the computational cost attributable specifically to our DPF framework.

#### Iterative sigma-clipping

We propose an iterative algorithm for determining a positive threshold, named ISC. The core concept of this algorithm is as follows: it assumes that the majority of data points are drawn from a single background normal distribution, while outliers on the right tail (i.e. potential positive samples) can bias the accurate estimation of the background distribution’s parameters. By iteratively removing these outliers and refitting the background distribution, the algorithm progressively converges to a stable, statistically significant threshold.

Algorithm steps:

1. Initialization:

Let the dataset be $X=\left\{{x}_1,{x}_2,\dots, {x}_n\right\}$. Set the significance level $\mathrm{\alpha}$ (e.g. 0.05), and accordingly calculate the upper quantile of the standard normal distribution $k={Z}_{1-\mathrm{\alpha} /2}$. Also, set a maximum number of iterations to prevent infinite looping.

1. Iteration process:

For iteration step $i=0,1,2,\dots$, perform the following operations:

a. Parameter Estimation: Based on the current dataset ${X}^{(i)}$ (where ${X}^{(0)}=X$ when $i=0$), calculate its sample mean ${\mathrm{\mu}}^{(i)}$ and sample standard deviation ${\mathrm{\sigma}}^{(i)}$.

b. Threshold Calculation: Calculate the temporary threshold for the current iteration:


$$ {t}^{(i)}={\mathrm{\mu}}^{(i)}+k\cdotp{\mathrm{\sigma}}^{(i)} $$

c. Outlier Removal (Clipping): Identify and remove all observations in the current dataset that are greater than the threshold ${t}^{(i)}$, considering them positive outliers. Form the dataset for the next iteration as:


$$ {X}^{\left(i+1\right)}=\left\{\mathrm{x}\in{X}^{(i)}\mid x\le{t}^{(i)}\right\} $$

1. Termination conditions:

The iteration stops when any of the following conditions is met:

a. The dataset ceases to change, i.e. ${X}^{\left(i+1\right)}={X}^{(i)}$.

b. The iteration count reaches the preset maximum $i=s$.

Let $T$ denote the final iteration step number.

1. Final threshold:

The final positive classification threshold is determined as:


$$ {t}_{final}={t}^{(T)}={\mathrm{\mu}}^{(T)}+k\cdotp{\mathrm{\sigma}}^{(T)} $$

Any observation in the original dataset $X$ satisfying $x>{t}_{final}$ can be considered a statistically significant positive sample at the significance level.

### Benchmarking based on deep mutational scanning datasets

To evaluate the performance of our proposed DPF, we validated our strategy using DMS datasets sourced from Livesey and Marsh’s study and Markin’s study [50, 51].

For each dataset, we predicted scores for all provided mutations across all nine DPF models. Subsequent analyses were conducted strictly within this scope; any mutations not present in the original dataset were excluded. (Note: The PafA dataset included only single-site glycine or valine substitutions, while the other datasets encompassed over 90% of all possible single-point mutations.)

As the datasets themselves lack a defined threshold for classifying positive mutations, we applied the ISC algorithm at multiple significance levels ($\alpha =0.1,\kern0.5em 0.05,\kern0.5em 0.01,\kern0.5em 0.001$) to determine the positivity threshold for the experimental DMS data. Using the same significance levels, we established corresponding prediction thresholds for the DPF scores (Supplementary Table S3). Results are not shown for cases where, at a given significance level, neither the dataset nor the DPF predictions yielded any values exceeding the calculated threshold (e.g. for InfA and PafA at $\alpha =0.001$).

This scenario constitutes a classic enrichment analysis. For a given DMS dataset and its corresponding DPF scores, after defining positive sets using the calculated thresholds, the overlap can be statistically evaluated using the hypergeometric distribution. The problem can be framed as follows: In a population of size $N$ (all measured mutations), there are $K$ true positive mutations. If we randomly select a sample of $n$ mutations (those predicted positive by DPF), the probability of observing exactly $k$ true positives in that sample is given by the hypergeometric probability mass function:


$$ P\left(X=k\right)=\frac{\left(\genfrac{}{}{0pt}{}{K}{k}\right)\left(\genfrac{}{}{0pt}{}{N-K}{n-k}\right)}{\left(\genfrac{}{}{0pt}{}{N}{n}\right)} $$

where $N$ is the total sample size of the DMS dataset, $K$ is the number of true positives within it, $n$ is the number of mutations predicted as positive by the DPF score, and $k$ is the number of mutations that are positive in both the DMS dataset and the DPF predictions.

Under the null hypothesis, the statistic follows a hypergeometric distribution:


$$ X\sim \mathrm{Hypergeometric}\left(N,K,n\right) $$

For enrichment significance, we calculate the right-tailed p-value:


$$ {p}_{\mathrm{enrichment}}=\boldsymbol{P}\left(\boldsymbol{X}\ge k\right)={\sum}_{i=k}^{\mathbf{\min}\left(n,\boldsymbol{K}\right)}\frac{\left(\genfrac{}{}{0pt}{}{\boldsymbol{K}}{i}\right)\left(\genfrac{}{}{0pt}{}{\boldsymbol{N}-\boldsymbol{K}}{n-i}\right)}{\left(\genfrac{}{}{0pt}{}{\boldsymbol{N}}{n}\right)} $$

For the specific calculation, we used the $stats. hypergeom. cdffunction$ in Python to compute the cumulative probability. The one-sided $P- value$ for the hypergeometric test is then given by $1- Cumulative Probabilit$.

In addition to comparisons with experimental data, we also calculated the enrichment performance of other alternative methods included in the dataset. To ensure comparability with the aforementioned DPF strategy, we selected the same number of top-scoring mutants for each method and used the proportion of true positive mutants within this set for horizontal comparison.

It is important to note that some methods assigned identical scores to a large number of mutants, and the corresponding gold-standard labels for these mutants were not uniformly positive or negative. To address this, we additionally calculated the mathematical expectation to determine the proportion, which could result in non-integer values (as shown in Supplementary data S4).

For the results across all DMS datasets, we used the average rank to comprehensively evaluate these methods. For any specific individual assessment, methods with identical results received the same rank. A lower average rank (closer to 1) indicates that the method demonstrated the best overall performance across multiple DMS datasets (Supplementary data S5).

### Strategy for *in silico* directed evolution of the *Blastobotrys adeninivorans* xanthine dehydrogenase molybdenum-pterin domain

We conducted a total of 12 rounds of *in silico* optimization for the *Ba*XD MOCO domain.

In the first round, we used the DPF-SSD_binding model to predict scores for all possible single-amino-acid mutants of the *Ba*XD MOCO Domain. We then performed an ADF test for a unit root sequentially on the ordered sequence of score differences. The position corresponding to the highest significance level was identified as the positive threshold. In the final round (Round 12), the scores were uniformly high, and we empirically set the threshold at 0.95 to select the top five mutants for experimental validation (approximately 40 000 mutants had scores above this threshold; Fig. 4b).

Mutants scoring above the threshold in a given round were paired to generate a combinatorial library of double mutants for the next round. This process—library generation, model scoring, and threshold calculation—was iterated (Fig. 4a).

Starting from round 3 to 7, we introduced a balancing strategy to increase the diversity of selected mutations, specifically to counter a strong bias towards cysteine (C) substitutions. We additionally included mutants above half the calculated threshold that contained a higher number of non-cysteine mutations to generate the subsequent round’s library.

Furthermore, from rounds 7 to 11, we performed a “Pan-single-mutation” scan on the top 2–4 candidate protein sequences from the previous round. This involved generating and scoring all possible single-amino-acid mutants for each of these parent sequences. These new single mutants were added to the candidate pool for the next round. The purpose of this step was to introduce new mutational information and prevent the search from being confined solely to combinations of mutations selected in the initial rounds (detailed evolutionary paths are provided in Supplementary data S2).

### Plasmid for transversion base editing

The base editing tool used in this study was derived from ABE8e (Addgene: #138489) by replacing the deaminase domain with a dehydrogenase. The backbone fragment was obtained by digesting ABE8e with restriction enzymes *NotI* and *BglII* (New England Biolabs). The dehydrogenase fragment was synthesized by Tianjin Zhonghe Gene Technology. The target fragment was amplified using Thermo Phusion High-Fidelity DNA Polymerase with 15-bp homology arms added to both ends, followed by homologous recombination using the Yeasen One-Step Cloning Kit. All transient expression vectors were transformed into *E. coli* DH5α competent cells and cultured at 37°C. Correct plasmids were obtained through monoclonal screening, small-scale plasmid extraction, restriction enzyme digestion validation, and Sanger sequencing.

### Transversion base editing in cell line

Cells were seeded in 48-well plates at ~40% confluency one day prior to transfection to ensure ~60% confluency after 18–24 h. A total of 750 ng of the base editing plasmid and 250 ng of single-guide RNA was transfected into HEK293T cells using 1.5 μL of Invitrogen Lipofectamine 2000 reagent, following the manufacturer’s protocol (Invitrogen Lipofectamine 3000 Kit). Three days post-transfection, genomic DNA was extracted: culture medium was removed, and 300 μL of cell lysis buffer I was added, followed by overnight incubation at 56°C. The lysate was then transferred to a 1.5 ml microcentrifuge tube, mixed with two volumes of absolute ethanol, and centrifuged to precipitate DNA.

### Amplicon library preparation

Targeted loci were analyzed via a two-step PCR amplification strategy. Primers were designed using Primer3web (https://primer3.ut.ee). Base editing efficiency was quantified using a previously reported somatic hypermutation analysis pipeline. First, paired-end sequencing reads were demultiplexed using the fastq-multx tool from ea-utils based on internal barcode sequences. Next, reads were aligned to the reference sequence using Bowtie2 to generate SAM files. Finally, statistical analysis of base substitutions, deletions, and insertions in the SAM files was performed to evaluate editing outcomes.

**Key Points**

- Computational methods are vital for directed evolution, but current strategies like PLM zero-shot approaches lack specificity priors. Their effectiveness is thus limited without experimental guidance.
- We introduce the Deep Prior Framework (DPF), which integrates structural plausibility with functional specificity priors through a novel Bernoulli-Attention (BATT) within Mixture of Experts (MoE) architecture to efficiently screen high-fitness mutants.
- On nine deep mutational scanning (DMS) datasets, DPF outperforms existing methods.
- Strikingly, fully *in silico* evolution of *Blastobotrys adeninivorans* xanthine dehydrogenase (*Ba*XD) without experimental feedback achieved an average activity enhancement of over four-fold, and a greater than nine-fold improvement in the top mutant.
- Furthermore, we applied DPF to annotate unreviewed sequences in UniProtKB. The framework assigned high-confidence functional labels to 38.9 million proteins (90.07% of a 42.9-million-sample subset).

## Supplementary Material

Supplementary_figure_bbag279

1_full_9_class_prot_info_bbag279

2_round_summary_bbag279

3_precision_recall_accuracy-test_bbag279

4_compare_DMS_Hit_0_001_bbag279

5_compare_DMS_ranking_0_001_bbag279

Authorship-Consent-and-Contribution-Statement-BIB-25-2562_R1_bbag279

## Data Availability

All data used for training DPF models are freely available from the public UniProt Knowledgebase (UniProtKB) repository, accessible at https://www.uniprot.org/. The nine DMS datasets are deposited in a public GitHub repository (https://github.com/ZhangSenxin/DPF).

## Code availability

All the code associated with this work open source in a public code repository (https://github.com/ZhangSenxin/DPF).
