# Supplementary material for: Deep Prior Framework: integrating functional specificity with general plausibility for targeted protein evolution
Source: Brief Bioinform. 2026 Jun 11;27(3):bbag279. doi: 10.1093/bib/bbag279 (PMC13256232; doi:10.1093/bib/bbag279)
Supplement: Supplementary_figure_bbag279 [file supplementary_figure_bbag279.pptx]

## Slide 1
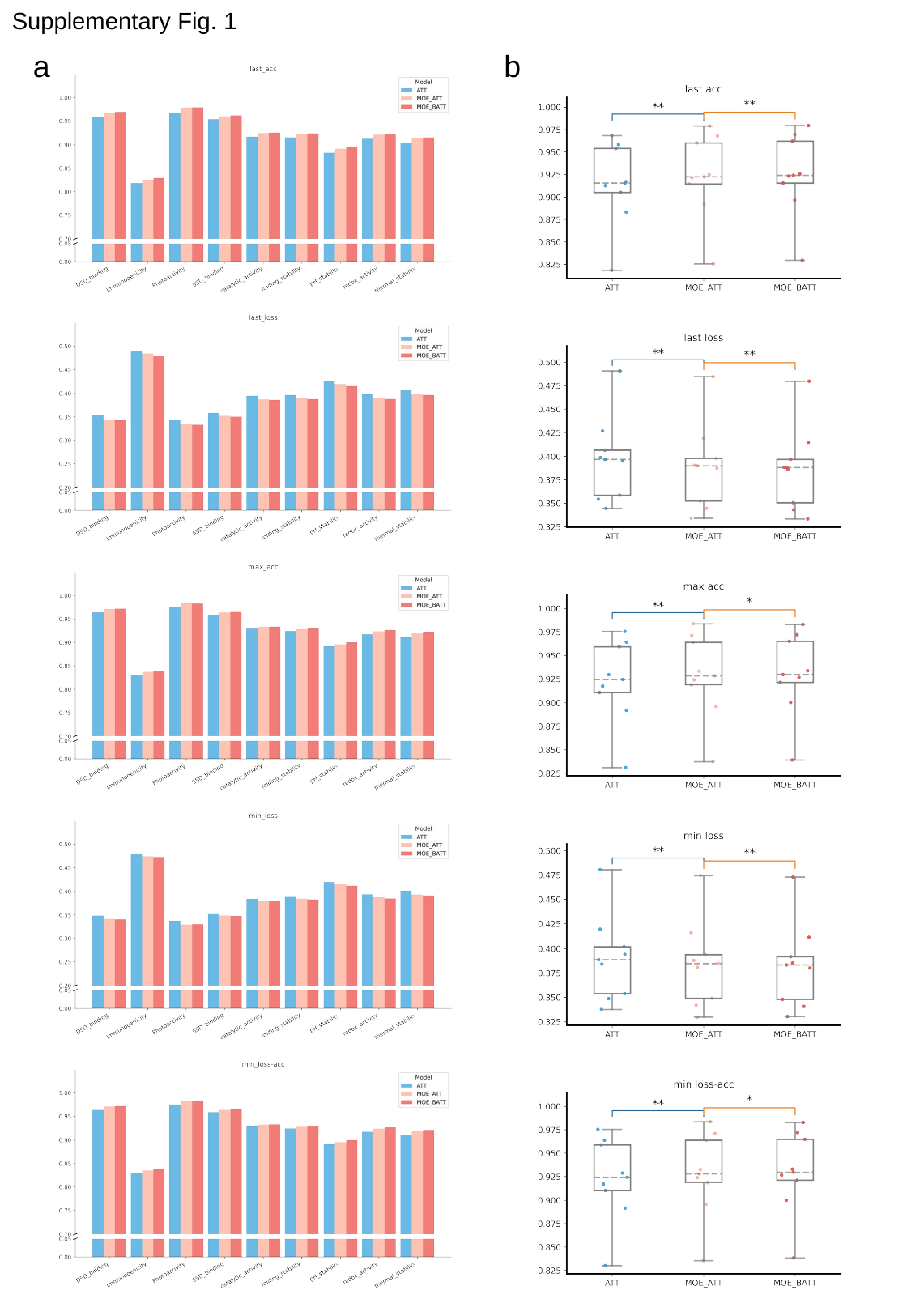

Supplementary Fig. 1
a
b

## Slide 2
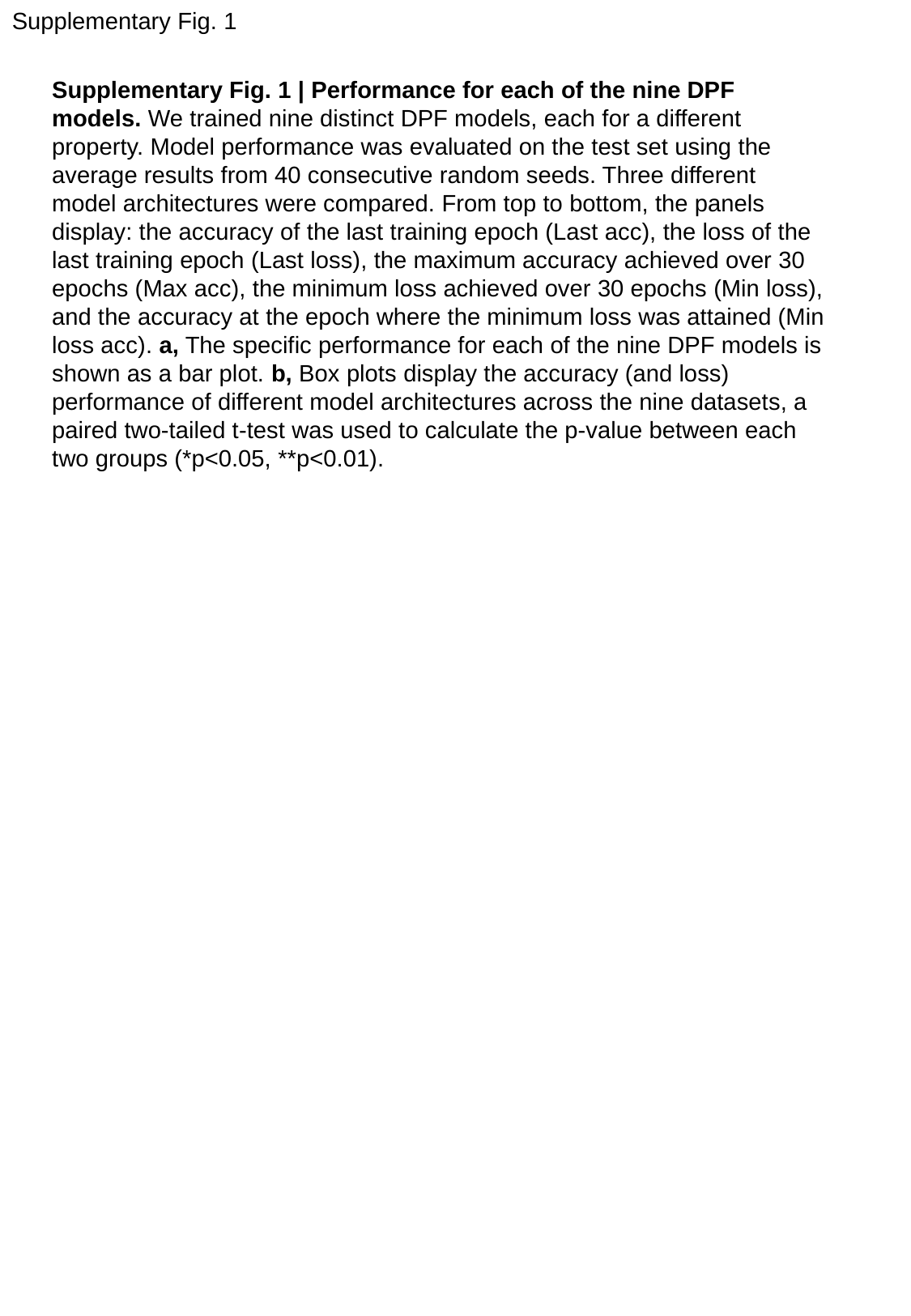

Supplementary Fig. 1
Supplementary Fig. 1 | Performance for each of the nine DPF models. We trained nine distinct DPF models, each for a different property. Model performance was evaluated on the test set using the average results from 40 consecutive random seeds. Three different model architectures were compared. From top to bottom, the panels display: the accuracy of the last training epoch (Last acc), the loss of the last training epoch (Last loss), the maximum accuracy achieved over 30 epochs (Max acc), the minimum loss achieved over 30 epochs (Min loss), and the accuracy at the epoch where the minimum loss was attained (Min loss acc). a, The specific performance for each of the nine DPF models is shown as a bar plot. b, Box plots​ display the accuracy (and loss)​ performance of different model architectures across the nine datasets, a paired two-tailed t-test was used to calculate the p-value between each two groups (*p<0.05, **p<0.01).

## Slide 3
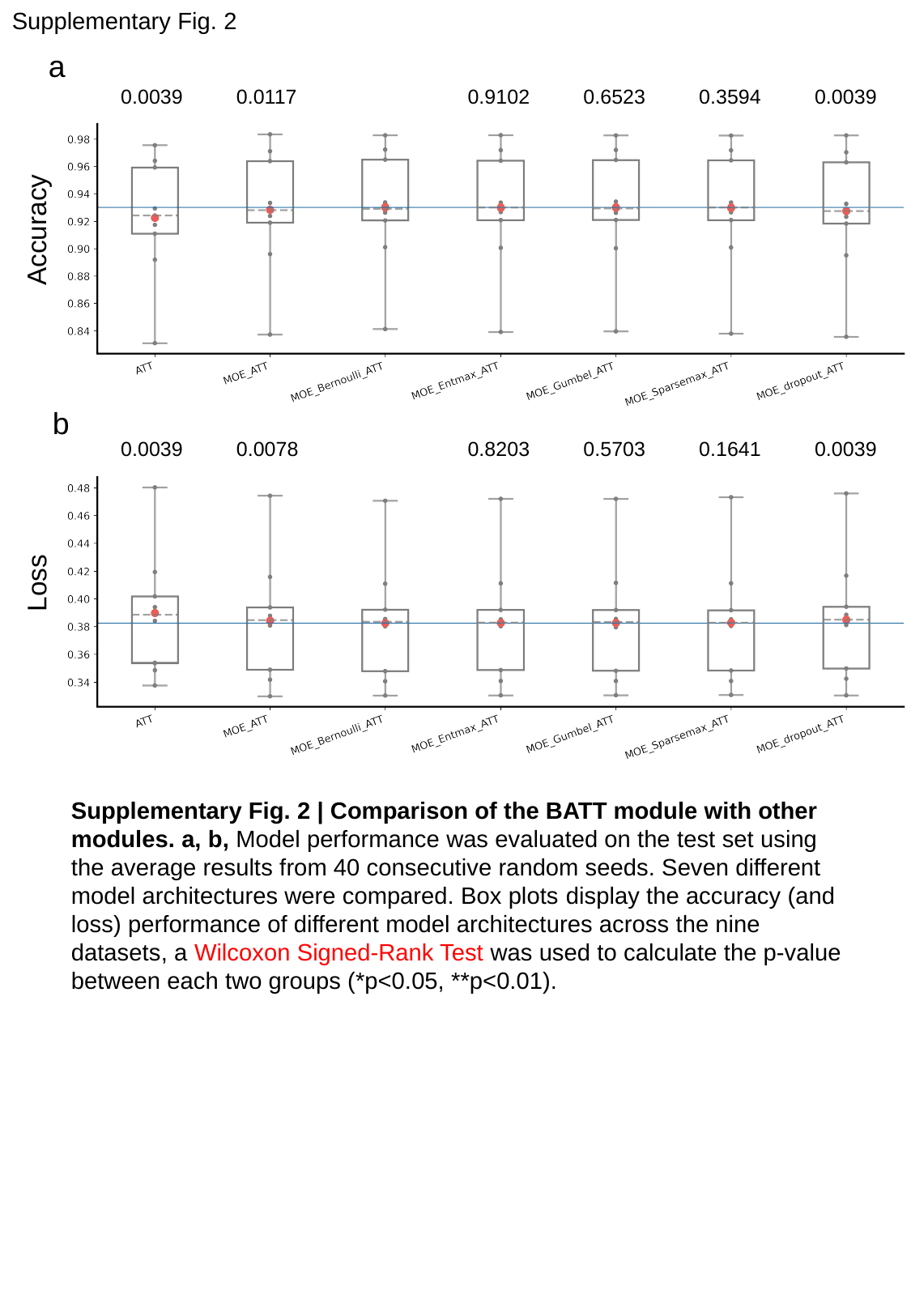

Supplementary Fig. 2
a
0.0039
0.0117
0.9102
0.6523
0.3594
0.0039
Accuracy
b
0.0039
0.0078
0.8203
0.5703
0.1641
0.0039
Loss
Supplementary Fig. 2 | Comparison of the BATT module with other modules. a, b, Model performance was evaluated on the test set using the average results from 40 consecutive random seeds. Seven different model architectures were compared. Box plots​ display the accuracy (and loss)​ performance of different model architectures across the nine datasets, a Wilcoxon Signed-Rank Test was used to calculate the p-value between each two groups (*p<0.05, **p<0.01).

## Slide 4
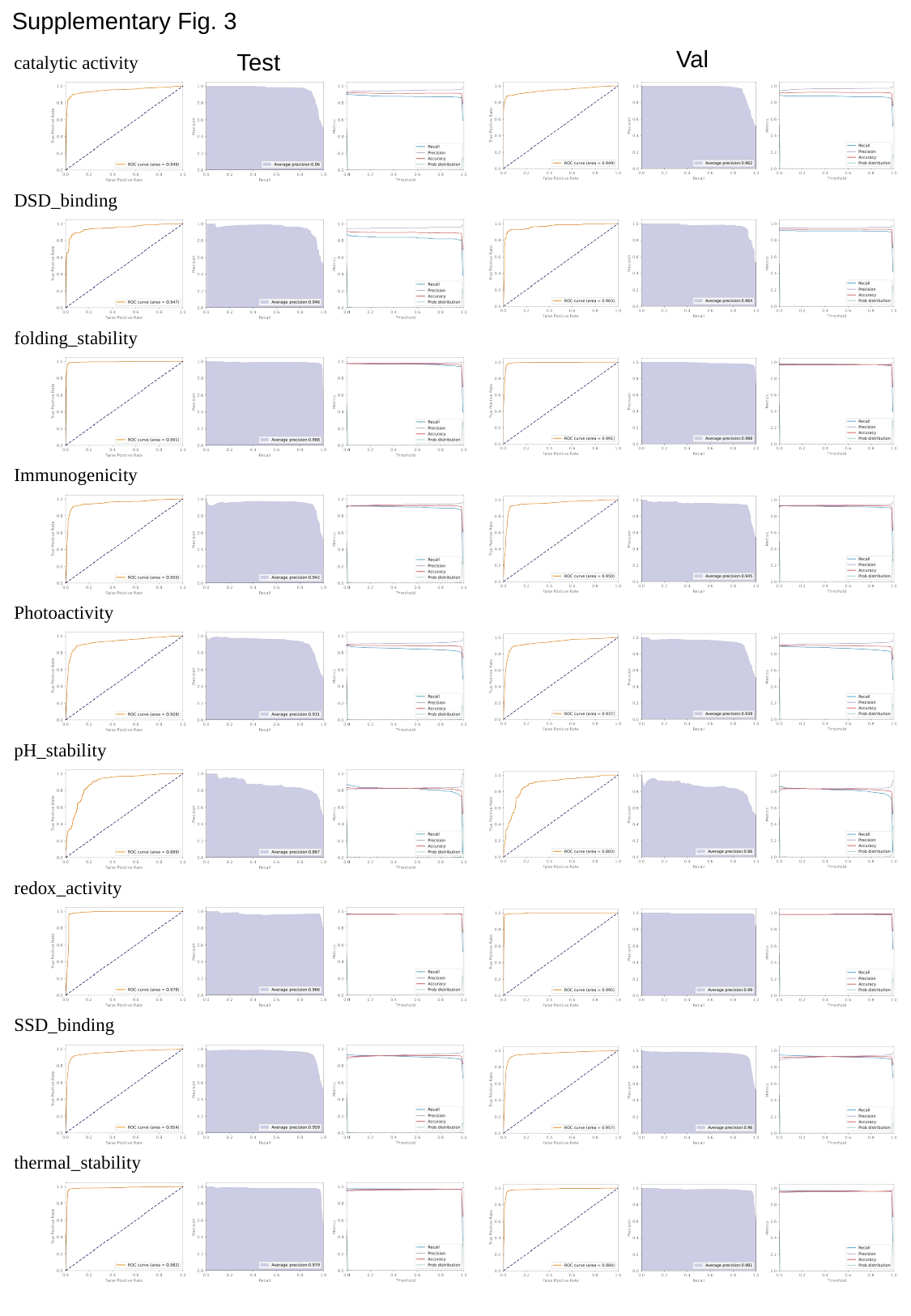

Supplementary Fig. 3
Val
Test
catalytic activity
DSD_binding
folding_stability
Immunogenicity
Photoactivity
pH_stability
redox_activity
SSD_binding
thermal_stability

## Slide 5
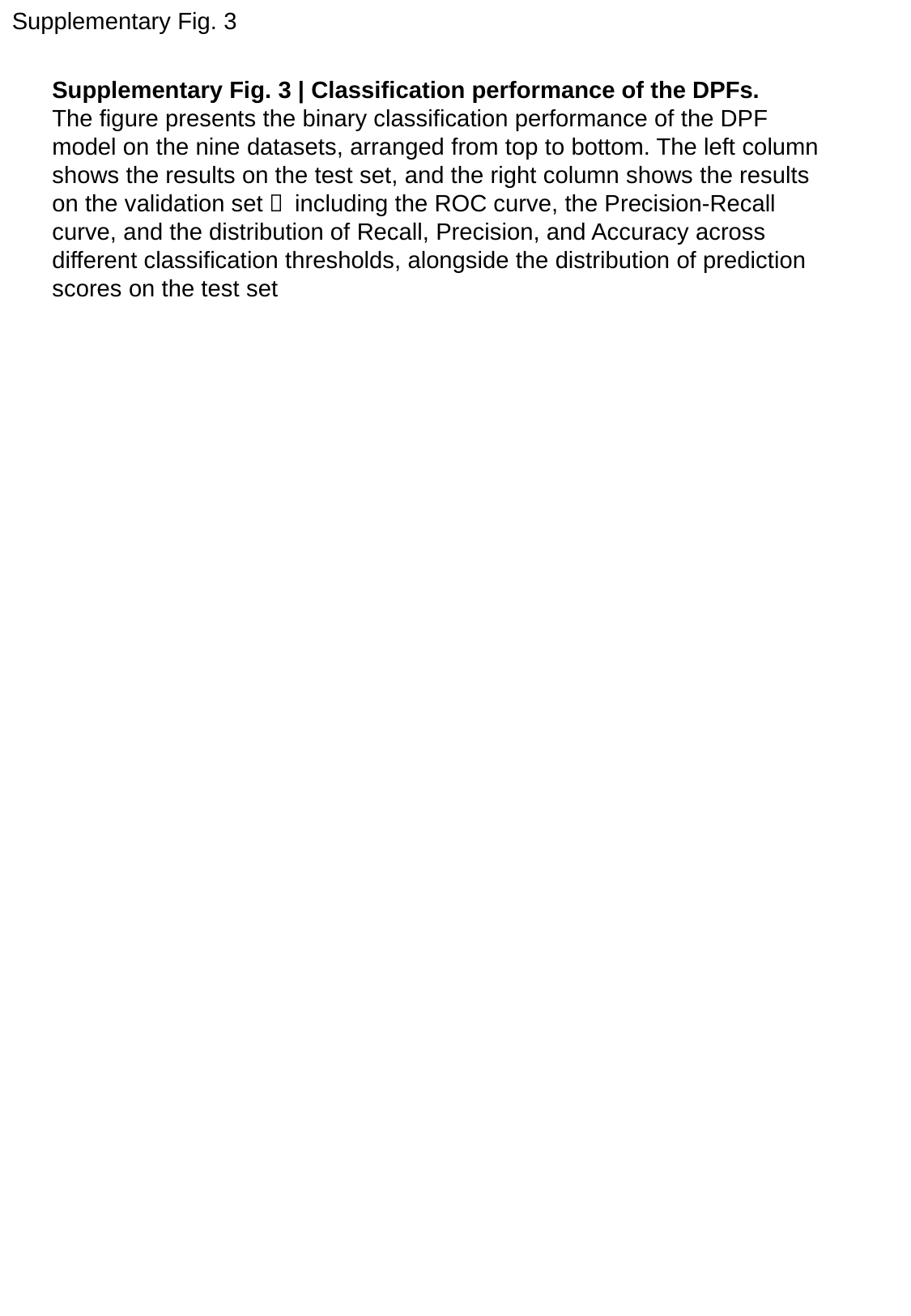

Supplementary Fig. 3
Supplementary Fig. 3 | Classification performance of the DPFs.
The figure presents the binary classification performance of the DPF model on the nine datasets, arranged from top to bottom. The left column shows the results on the test set, and the right column shows the results on the validation set， including the ROC curve, the Precision-Recall curve, and the distribution of Recall, Precision, and Accuracy across different classification thresholds, alongside the distribution of prediction scores on the test set

## Slide 6
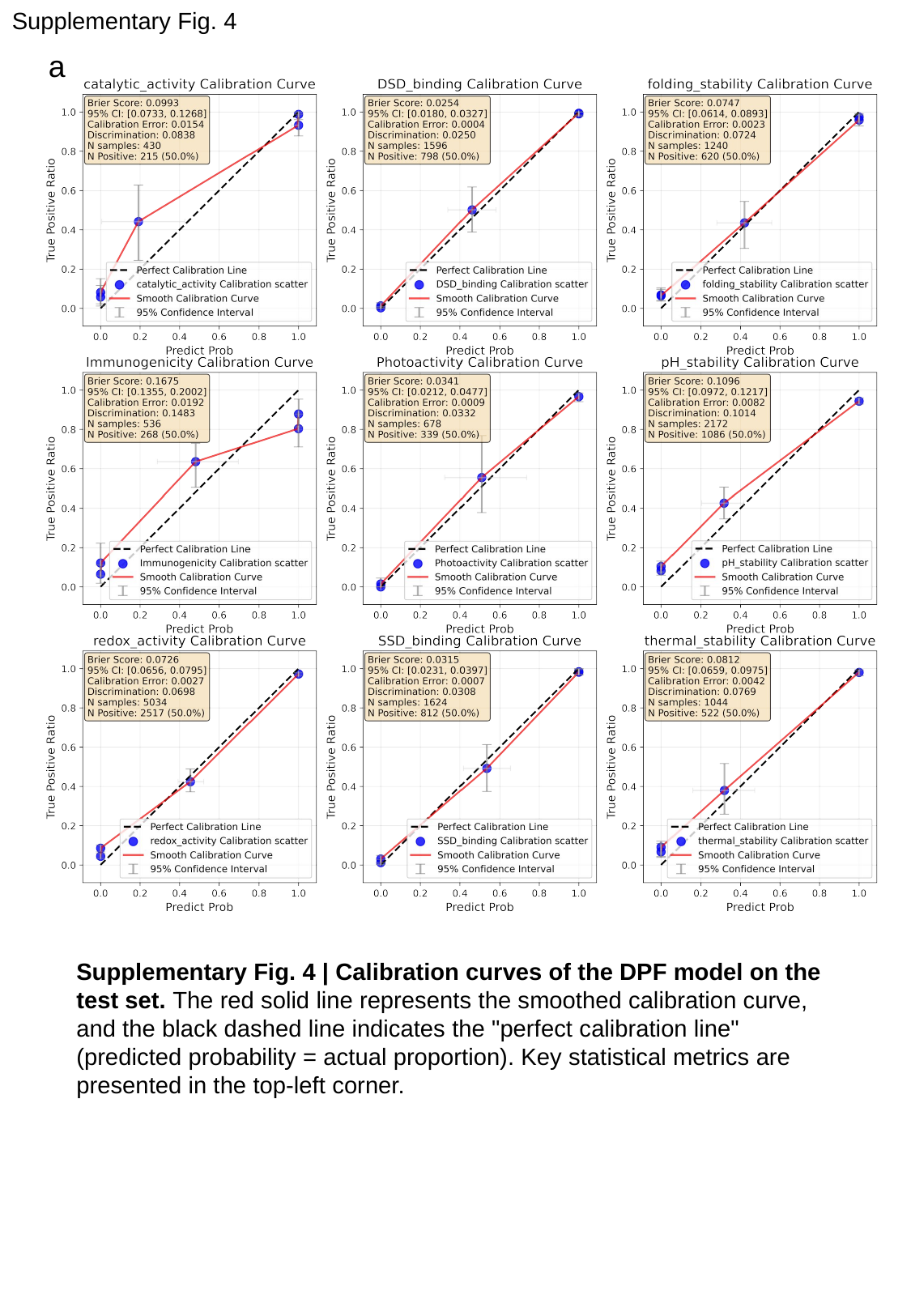

Supplementary Fig. 4
a
Supplementary Fig. 4 | Calibration curves of the DPF model on the test set. The red solid line represents the smoothed calibration curve, and the black dashed line indicates the "perfect calibration line" (predicted probability = actual proportion). Key statistical metrics are presented in the top-left corner.

## Slide 7
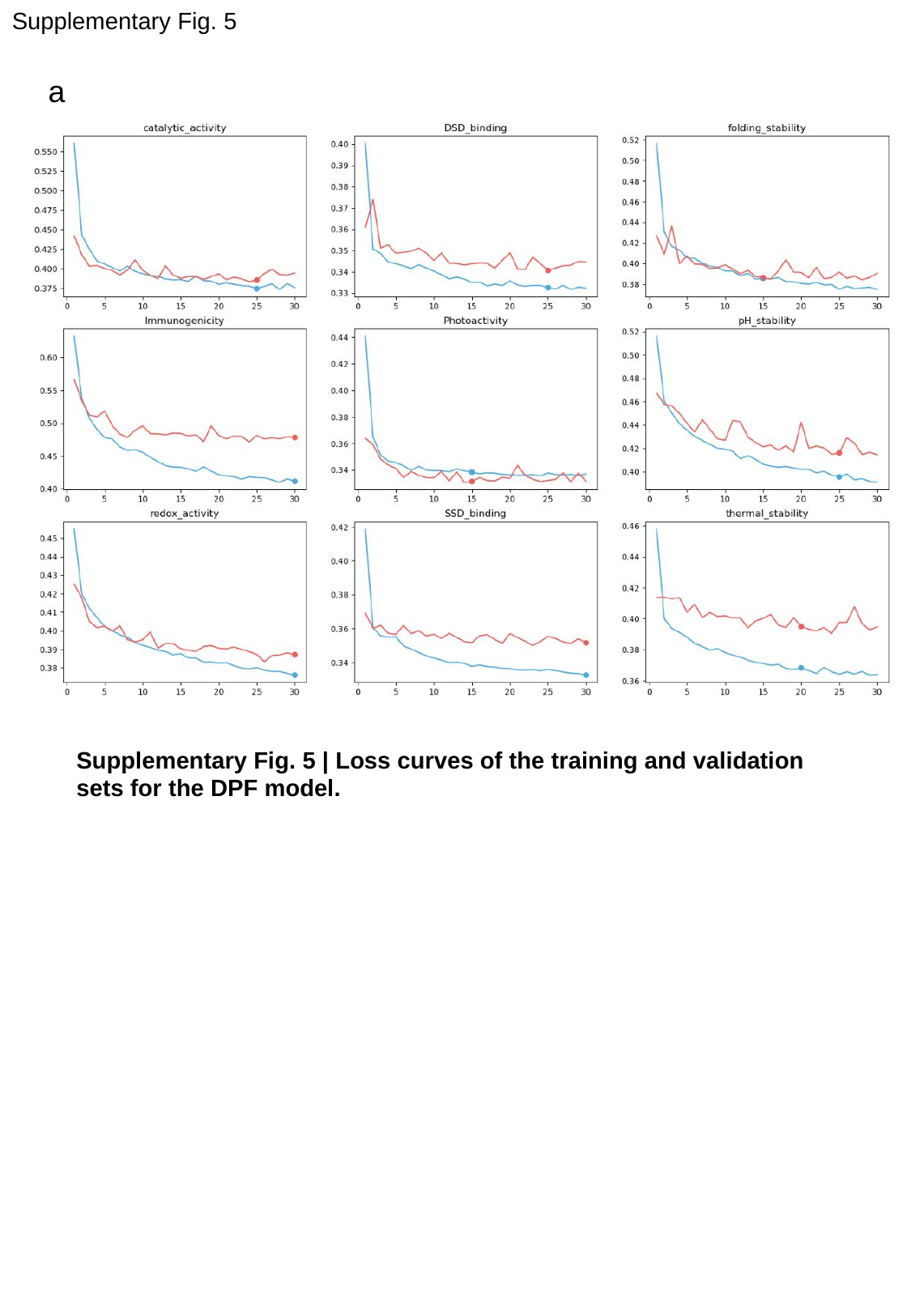

Supplementary Fig. 5
a
Supplementary Fig. 5 | Loss curves of the training and validation sets for the DPF model.

## Slide 8
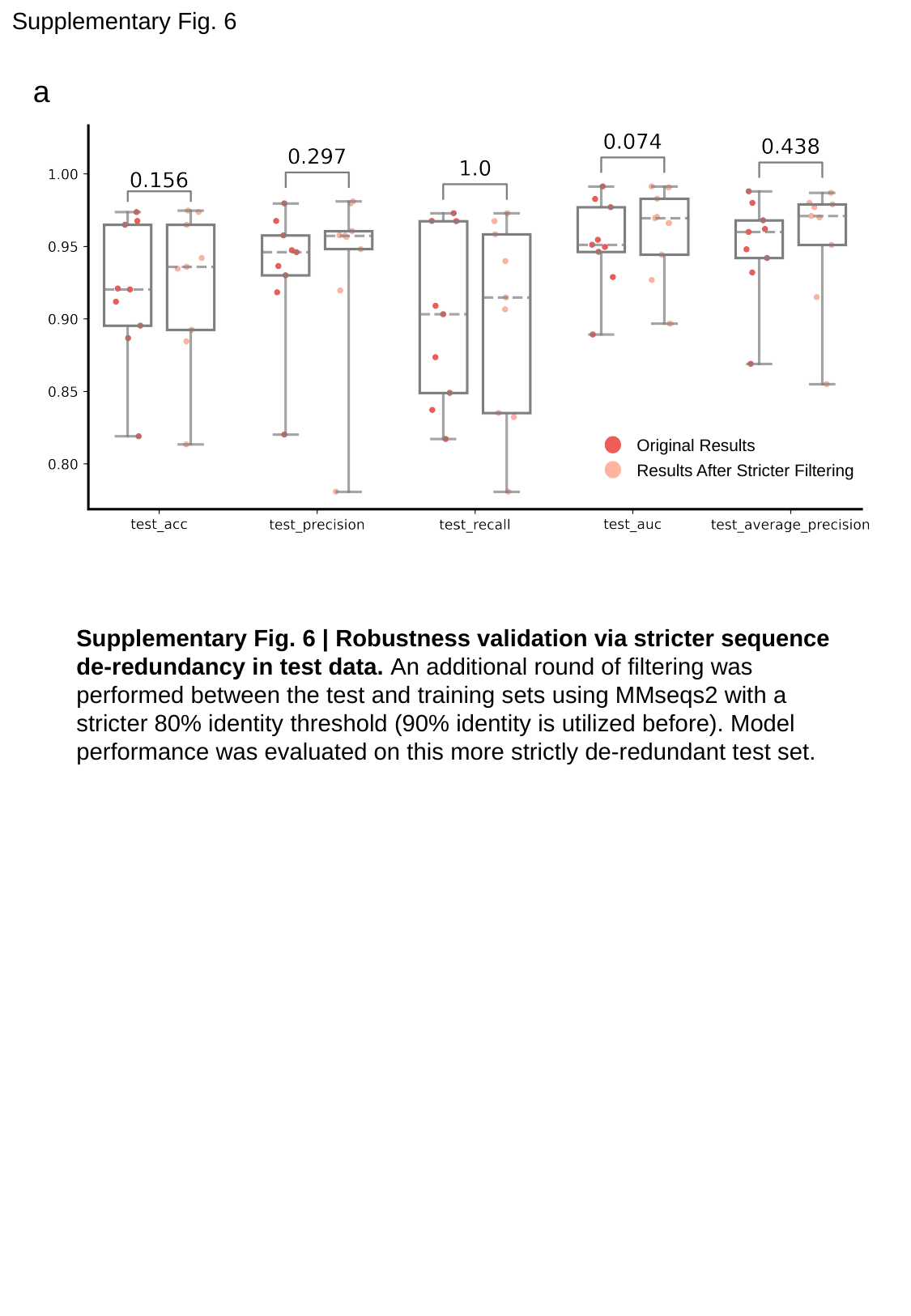

Supplementary Fig. 6
a
Original Results
Results After Stricter Filtering
Supplementary Fig. 6 | Robustness validation via stricter sequence de-redundancy in test data. An additional round of filtering was performed between the test and training sets using MMseqs2 with a stricter 80% identity threshold (90% identity is utilized before). Model performance was evaluated on this more strictly de-redundant test set.

## Slide 9
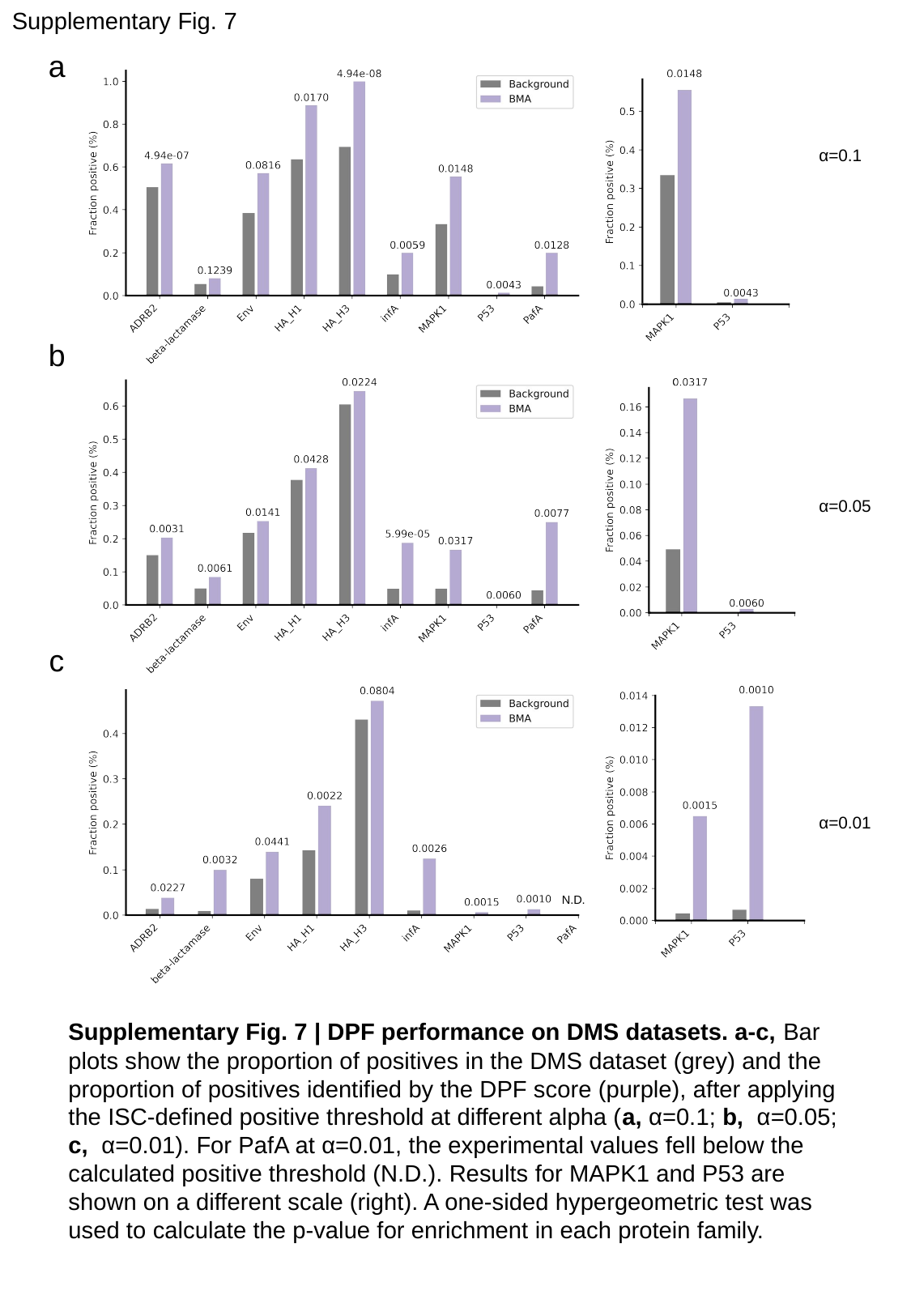

Supplementary Fig. 7
a
α=0.1
b
α=0.05
c
N.D.
α=0.01
Supplementary Fig. 7 | DPF performance on DMS datasets. a-c, Bar plots show the proportion of positives in the DMS dataset (grey) and the proportion of positives identified by the DPF score (purple), after applying the ISC-defined positive threshold at different alpha (a, α=0.1; b, α=0.05; c, α=0.01). For PafA at α=0.01, the experimental values fell below the calculated positive threshold (N.D.). Results for MAPK1 and P53 are shown on a different scale (right). A one-sided hypergeometric test was used to calculate the p-value for enrichment in each protein family.

## Slide 10
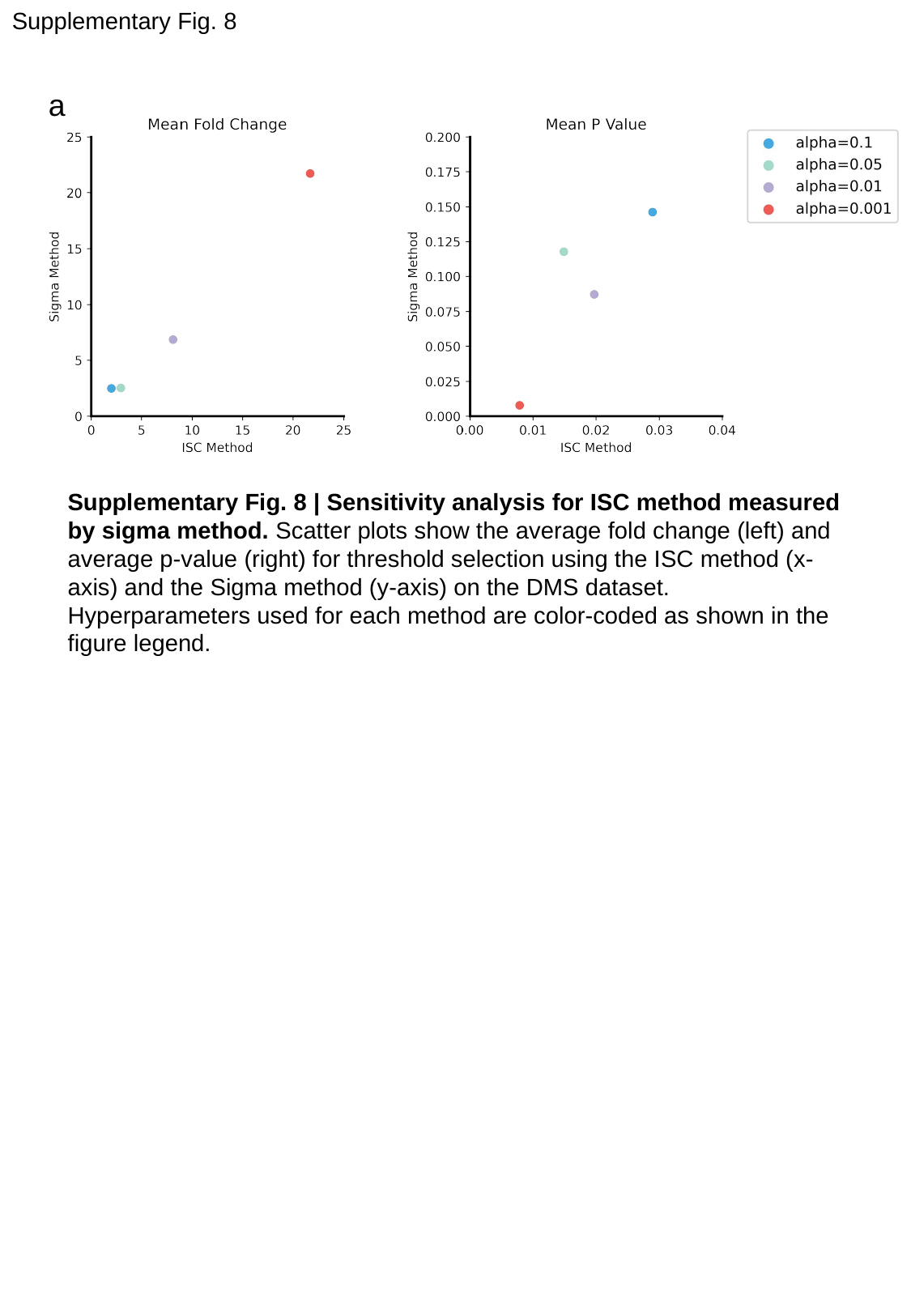

Supplementary Fig. 8
a
Supplementary Fig. 8 | Sensitivity analysis for ISC method measured by sigma method. Scatter plots show the average fold change (left) and average p-value (right) for threshold selection using the ISC method (x-axis) and the Sigma method (y-axis) on the DMS dataset. Hyperparameters used for each method are color-coded as shown in the figure legend.

## Slide 11
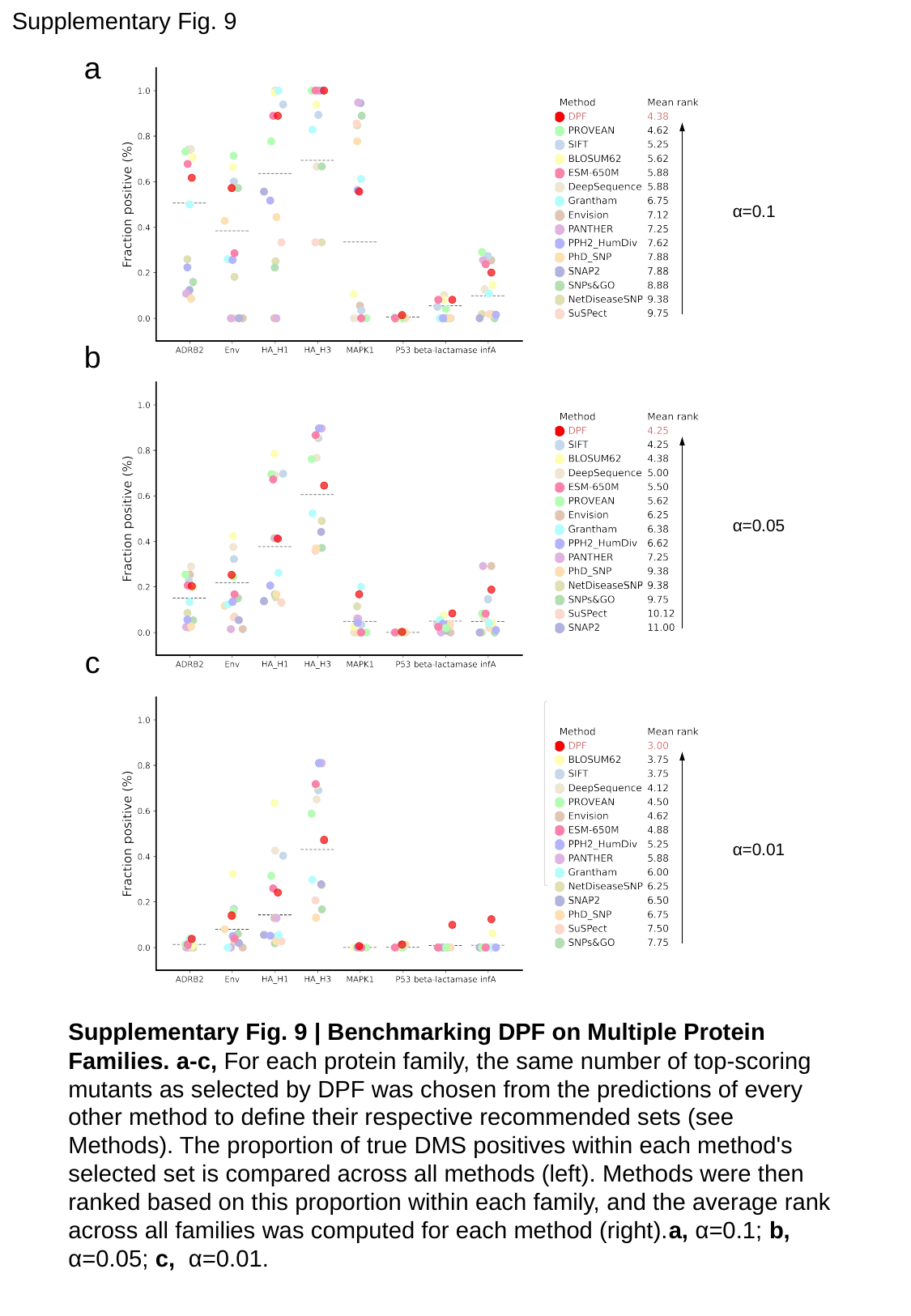

Supplementary Fig. 9
a
α=0.1
b
α=0.05
c
α=0.01
Supplementary Fig. 9 | Benchmarking DPF on Multiple Protein Families. a-c, For each protein family, the same number of top-scoring mutants as selected by DPF was chosen from the predictions of every other method to define their respective recommended sets (see Methods). The proportion of true DMS positives within each method's selected set is compared across all methods (left). Methods were then ranked based on this proportion within each family, and the average rank across all families was computed for each method (right).a, α=0.1; b, α=0.05; c, α=0.01.

## Slide 12
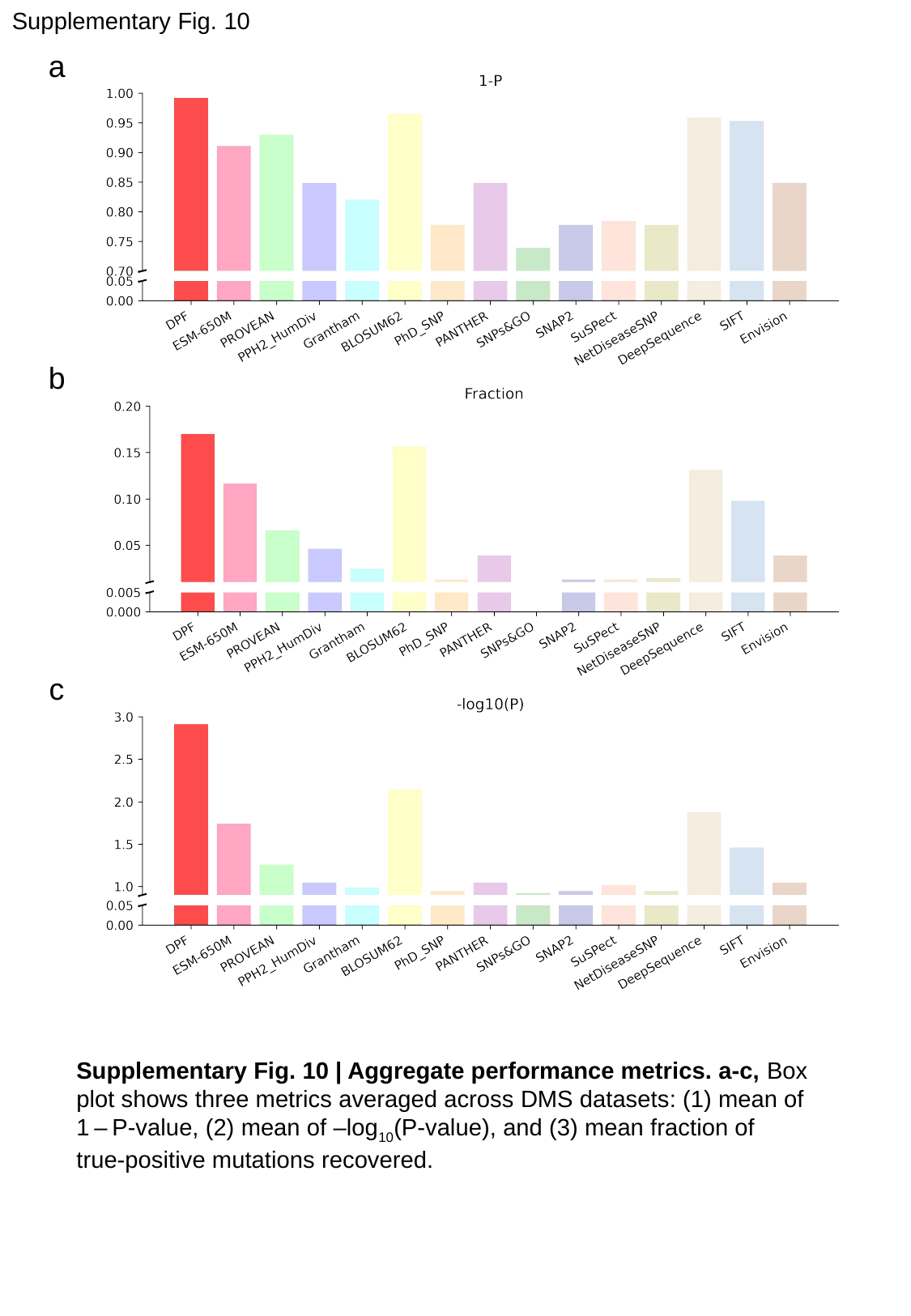

Supplementary Fig. 10
a
b
c
Supplementary Fig. 10 | Aggregate performance metrics. a-c, Box plot shows three metrics averaged across DMS datasets: (1) mean of 1 – P-value, (2) mean of –log10(P-value), and (3) mean fraction of true‑positive mutations recovered.

## Slide 13
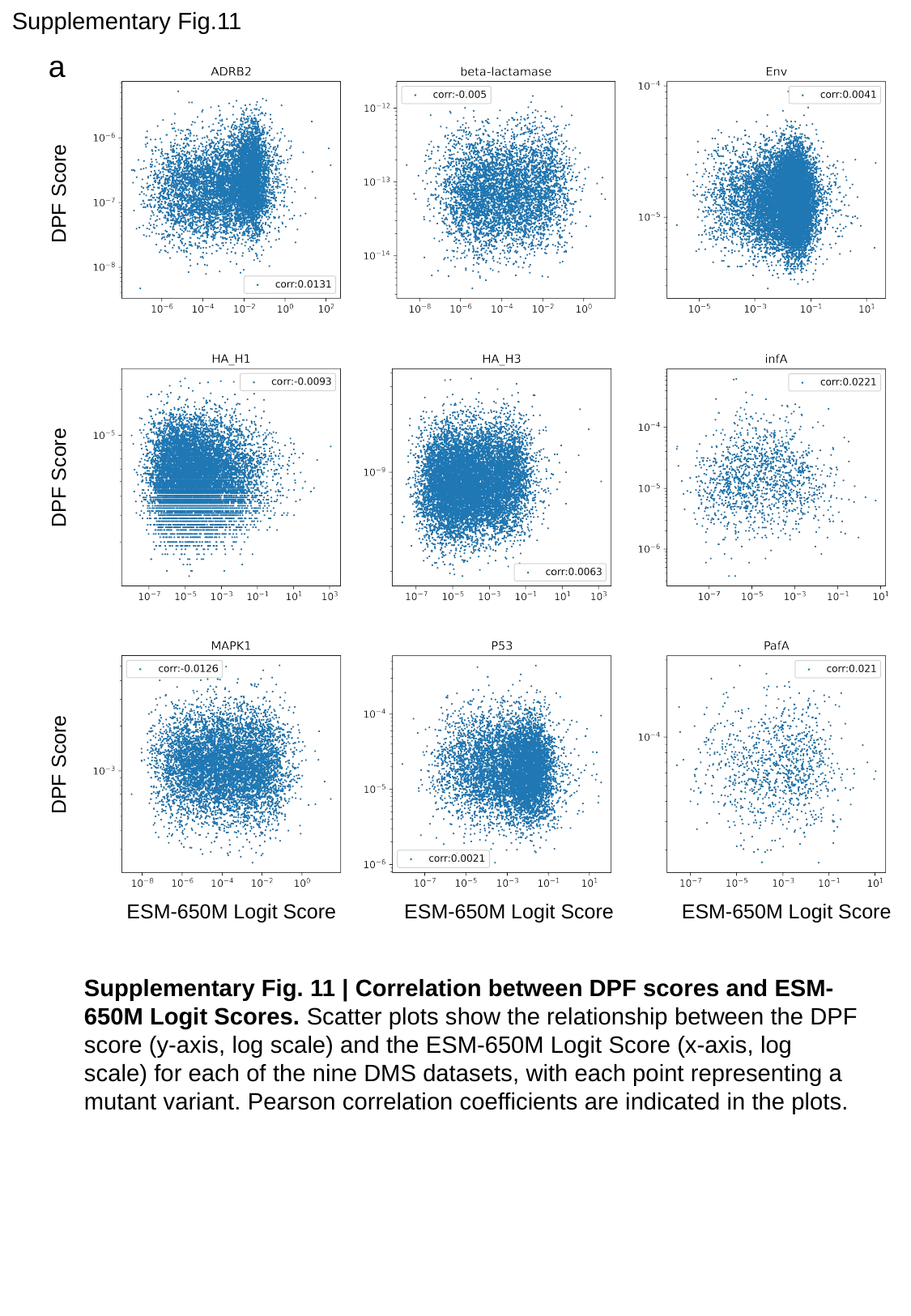

Supplementary Fig.11
a
DPF Score
DPF Score
DPF Score
ESM-650M Logit Score
ESM-650M Logit Score
ESM-650M Logit Score
Supplementary Fig. 11 | Correlation between DPF scores and ESM-650M Logit Scores. Scatter plots show the relationship between the DPF score (y-axis, log scale) and the ESM-650M Logit Score (x-axis, log scale) for each of the nine DMS datasets, with each point representing a mutant variant. Pearson correlation coefficients are indicated in the plots.

## Slide 14
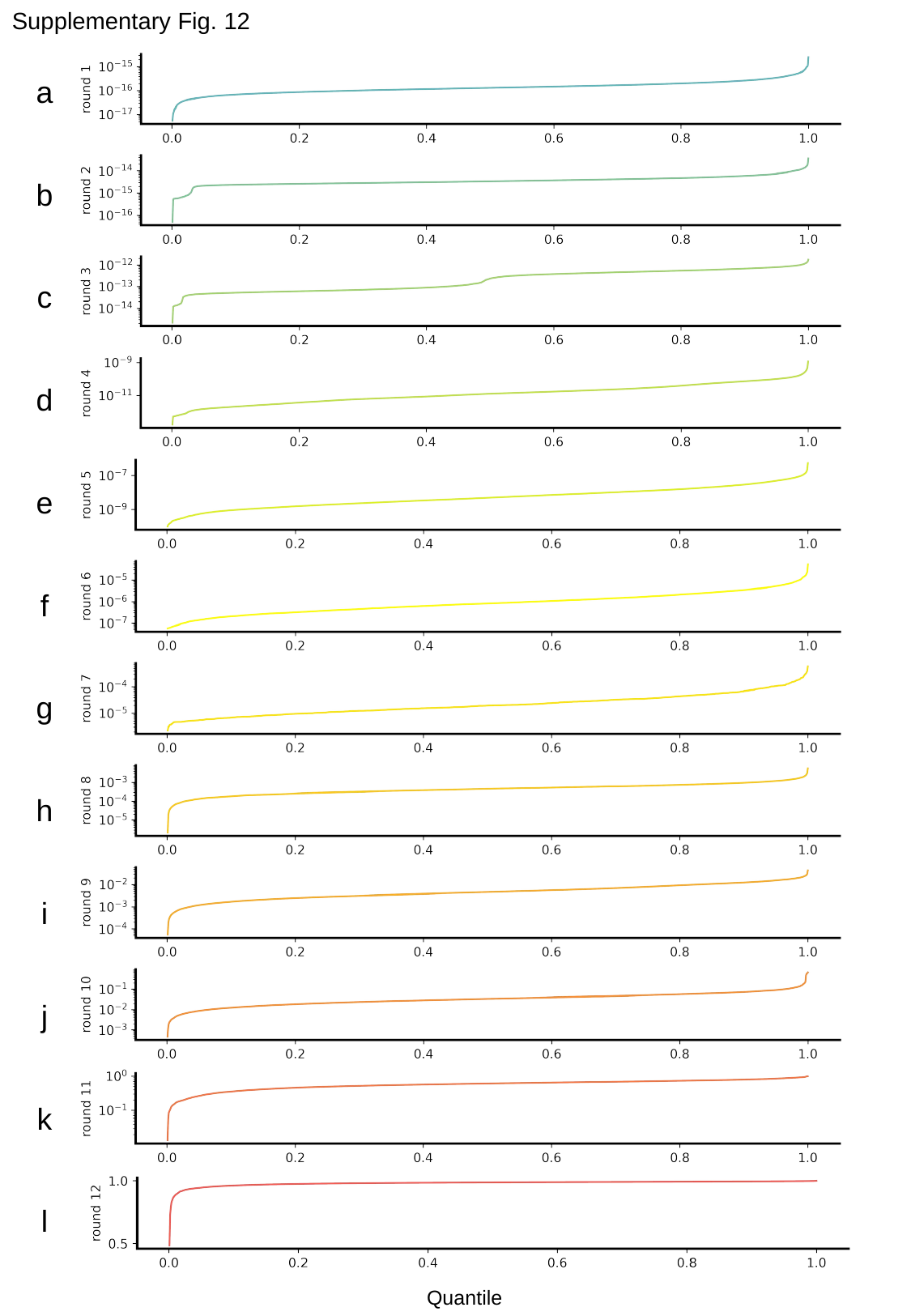

Supplementary Fig. 12
a
b
c
d
e
f
g
h
i
j
k
l
Quantile

## Slide 15
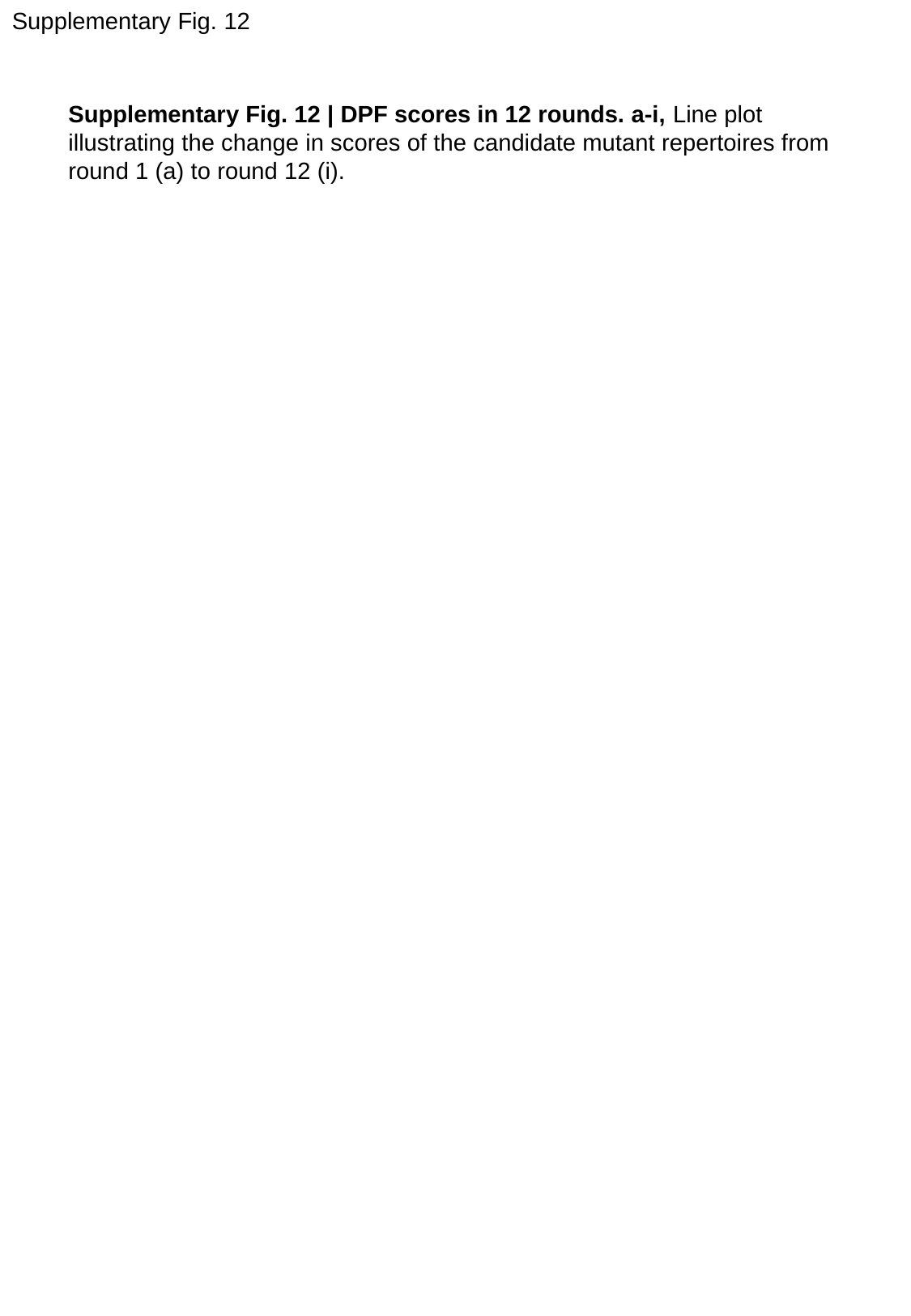

Supplementary Fig. 12
Supplementary Fig. 12 | DPF scores in 12 rounds. a-i, Line plot illustrating the change in scores of the candidate mutant repertoires from round 1 (a) to round 12 (i).

## Slide 16
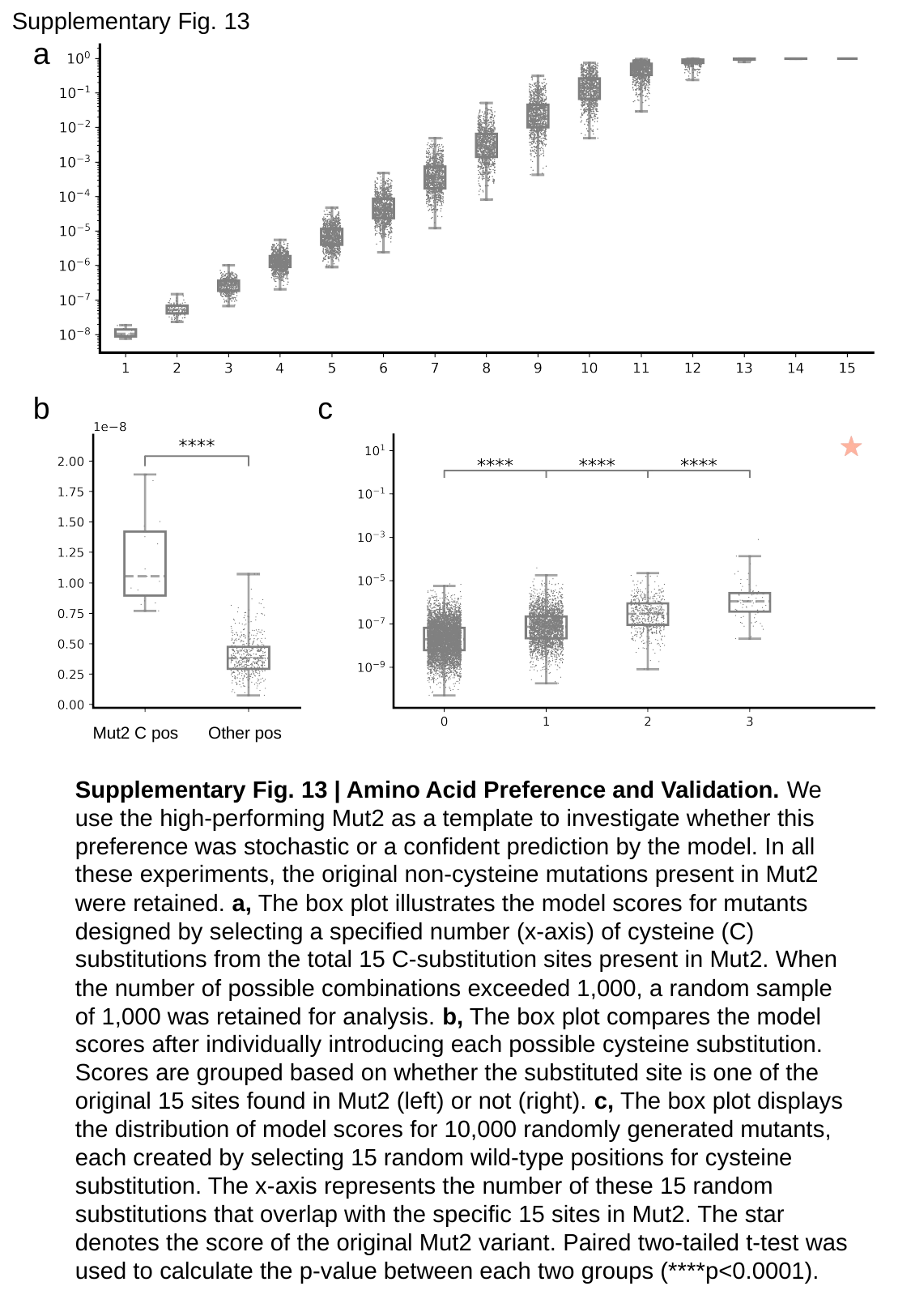

Supplementary Fig. 13
a
b
c
Mut2 C pos
Other pos
Supplementary Fig. 13 | Amino Acid Preference and Validation. We use the high-performing Mut2 as a template to investigate whether this preference was stochastic or a confident prediction by the model. In all these experiments, the original non-cysteine mutations present in Mut2 were retained. a,​ The box plot illustrates the model scores for mutants designed by selecting a specified number (x-axis) of cysteine (C) substitutions from the total 15 C-substitution sites present in Mut2. When the number of possible combinations exceeded 1,000, a random sample of 1,000 was retained for analysis. b,​ The box plot compares the model scores after individually introducing each possible cysteine substitution. Scores are grouped based on whether the substituted site is one of the original 15 sites found in Mut2 (left) or not (right). c,​ The box plot displays the distribution of model scores for 10,000 randomly generated mutants, each created by selecting 15 random wild-type positions for cysteine substitution. The x-axis represents the number of these 15 random substitutions that overlap with the specific 15 sites in Mut2. The star denotes the score of the original Mut2 variant. Paired two-tailed t-test was used to calculate the p-value between each two groups (****p<0.0001).

## Slide 17
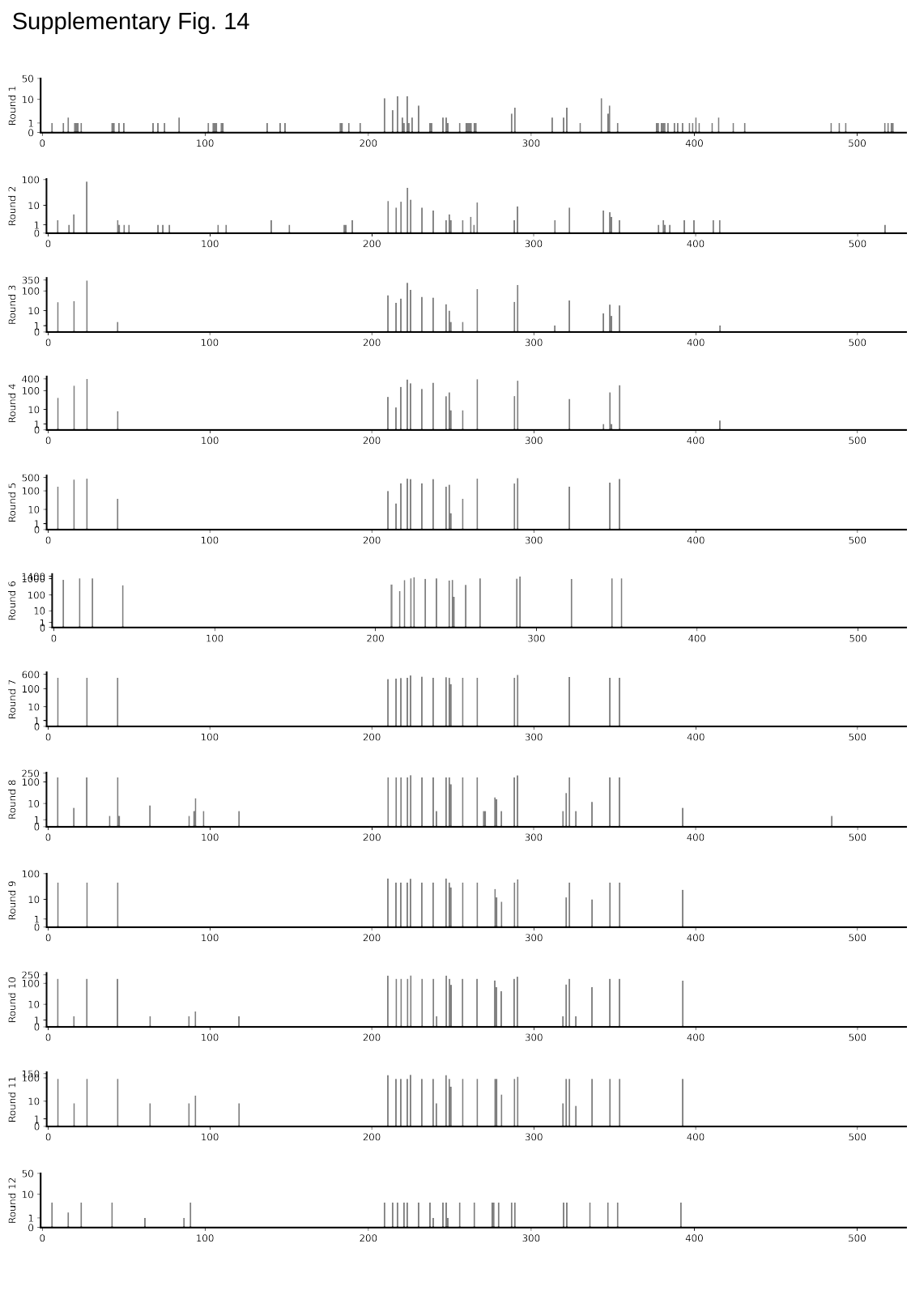

Supplementary Fig. 14

## Slide 18
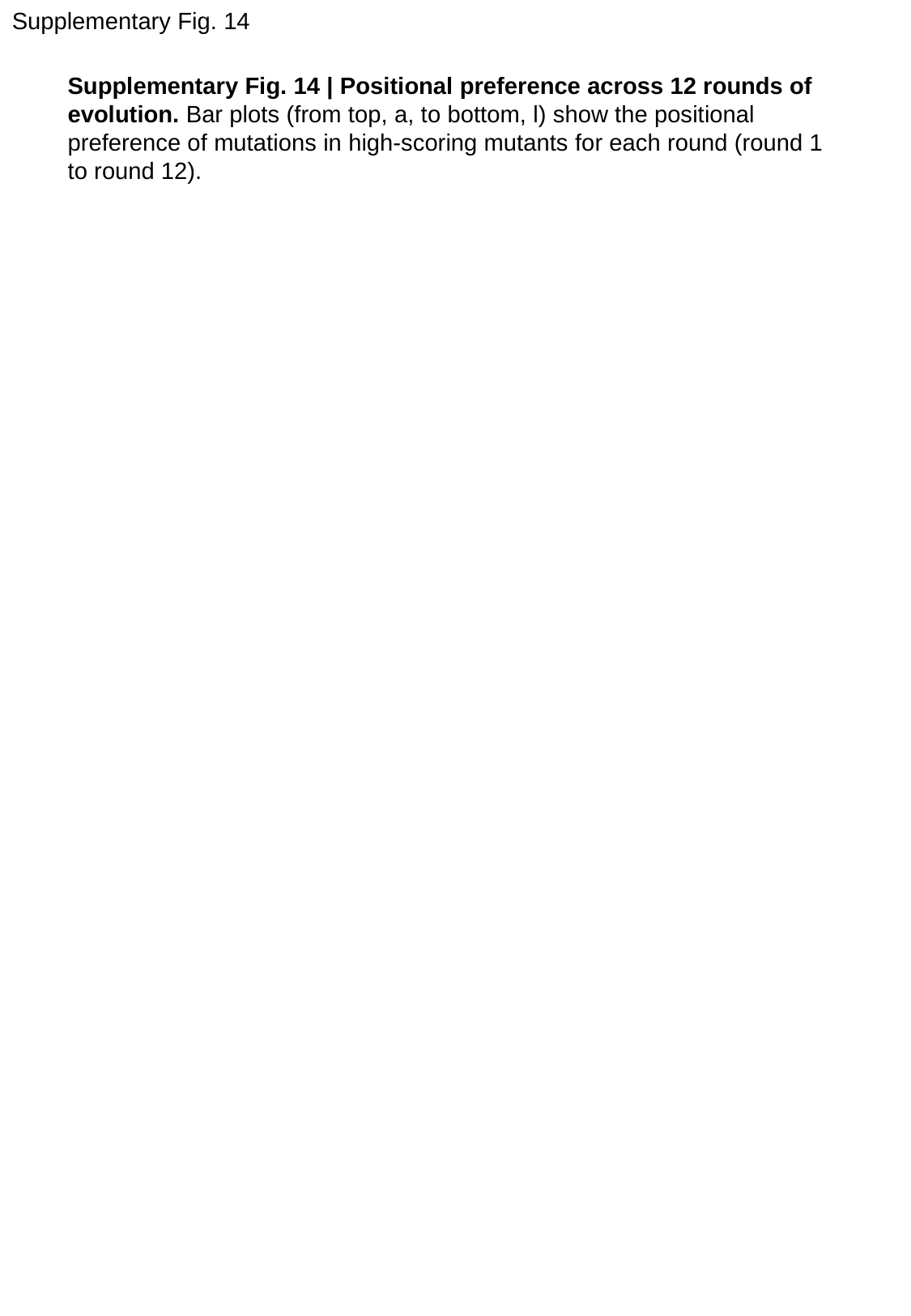

Supplementary Fig. 14
Supplementary Fig. 14 | Positional preference across 12 rounds of evolution.​ Bar plots (from top, a, to bottom, l) show the positional preference of mutations in high-scoring mutants for each round (round 1 to round 12).

## Slide 19
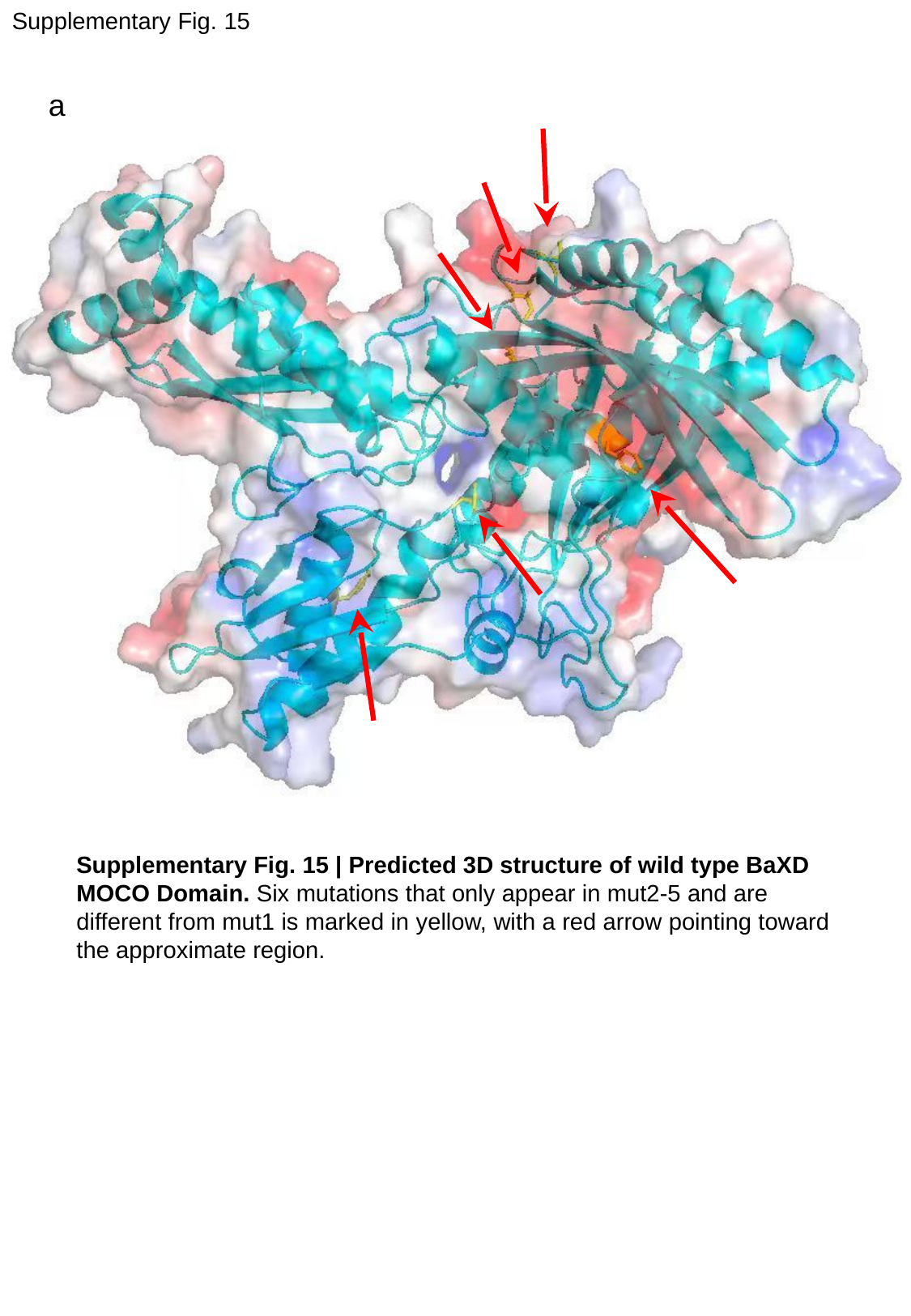

Supplementary Fig. 15
a
Supplementary Fig. 15 | Predicted 3D structure of wild type BaXD MOCO Domain. Six mutations that only appear in mut2-5 and are different from mut1 is marked in yellow, with a red arrow pointing toward the approximate region.

## Slide 20
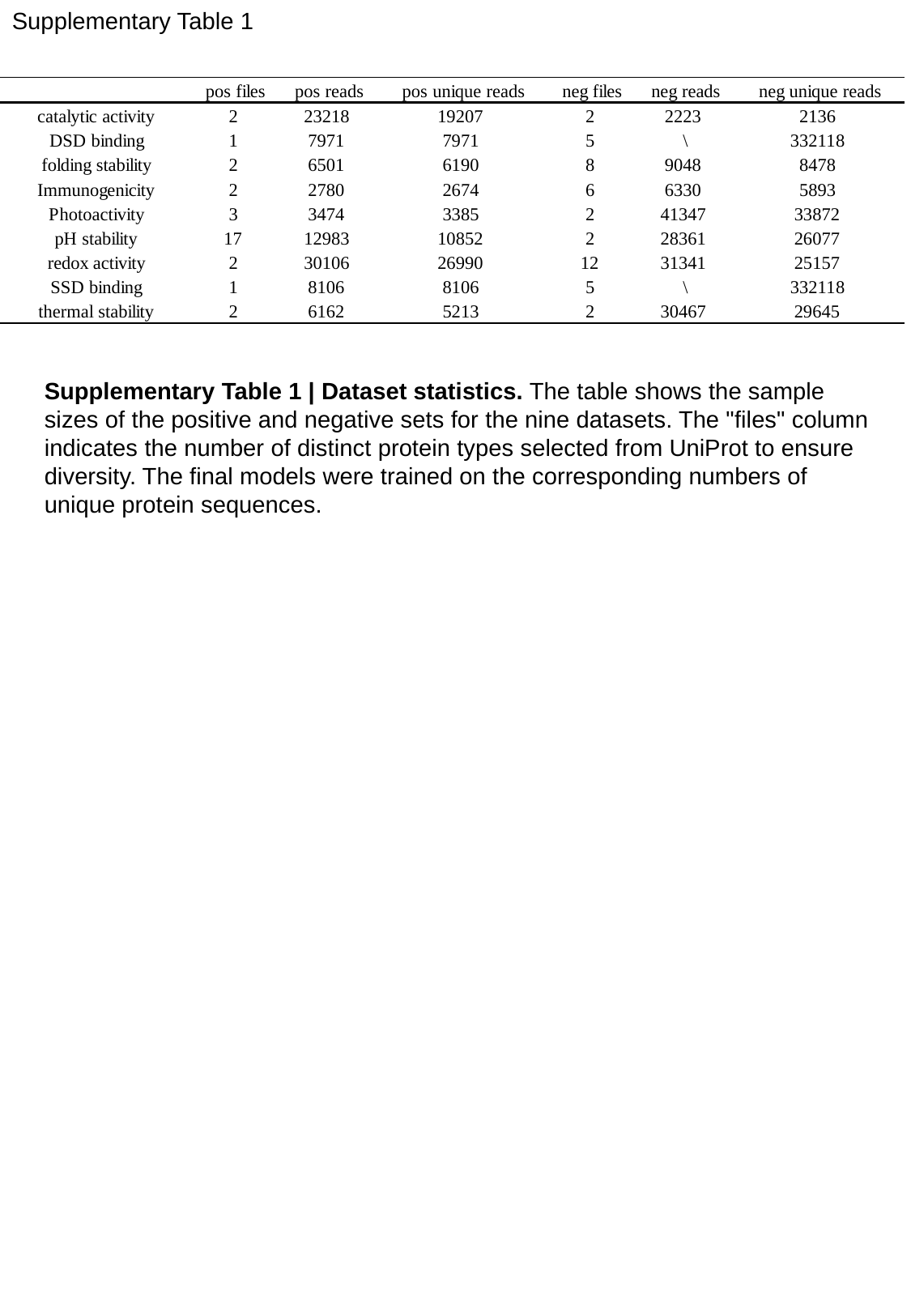

Supplementary Table 1
Supplementary Table 1 | Dataset statistics.​ The table shows the sample sizes of the positive and negative sets for the nine datasets. The "files" column indicates the number of distinct protein types selected from UniProt to ensure diversity. The final models were trained on the corresponding numbers of unique protein sequences.

## Slide 21
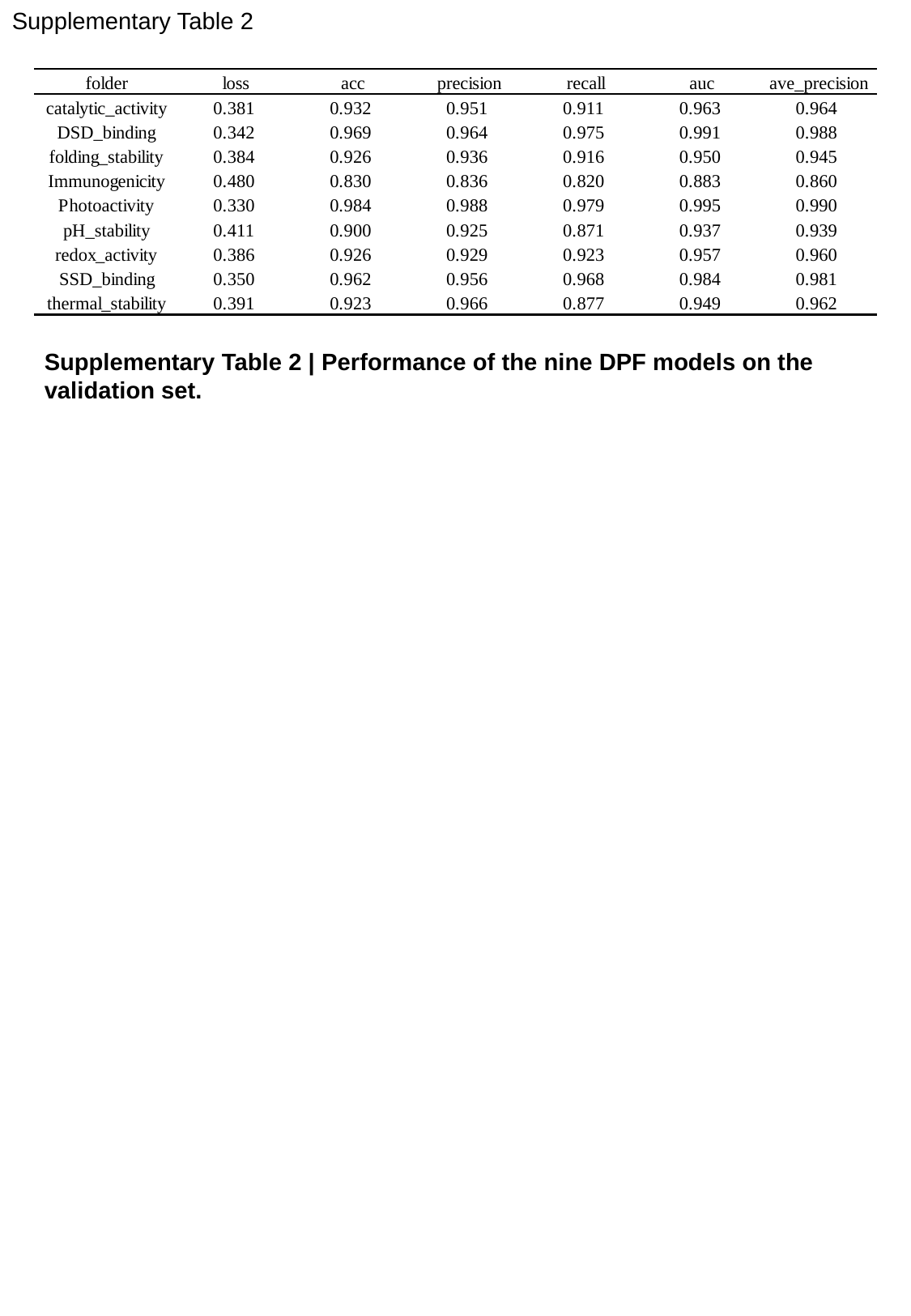

Supplementary Table 2
Supplementary Table 2 | Performance of the nine DPF models on the validation set.

## Slide 22
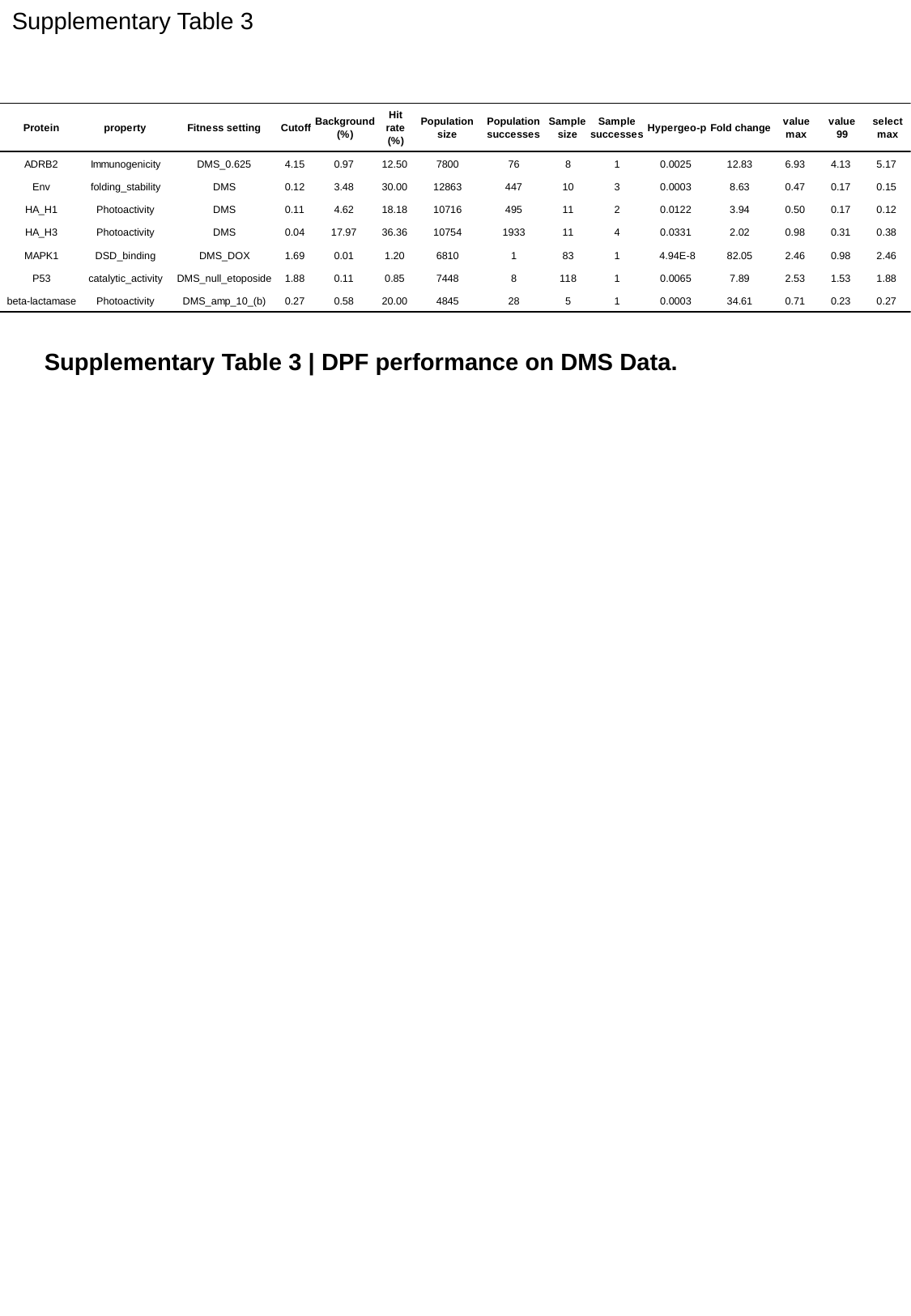

Supplementary Table 3
Supplementary Table 3 | DPF performance on DMS Data.

## Slide 23
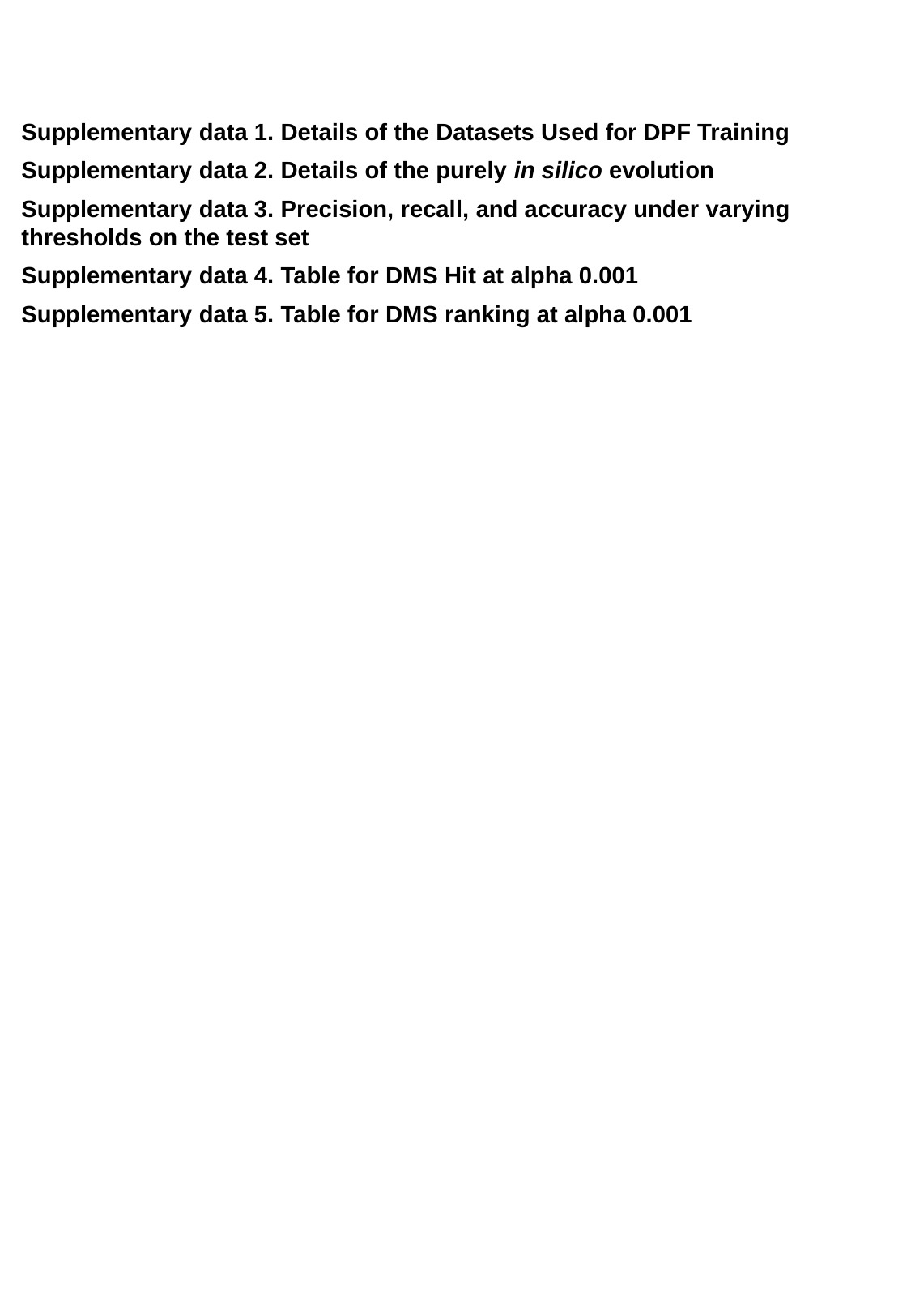

Supplementary data 1. Details of the Datasets Used for DPF Training
Supplementary data 2. Details of the purely in silico evolution
Supplementary data 3. Precision, recall, and accuracy under varying thresholds on the test set
Supplementary data 4. Table for DMS Hit at alpha 0.001
Supplementary data 5. Table for DMS ranking at alpha 0.001
