# Supplementary material for: Deep Prior Framework: integrating functional specificity with general plausibility for targeted protein evolution
Source: Brief Bioinform. 2026 Jun 11;27(3):bbag279. doi: 10.1093/bib/bbag279 (PMC13256232; doi:10.1093/bib/bbag279)
Supplement: Authorship-Consent-and-Contribution-Statement-BIB-25-2562_R1_bbag279 [file authorship-consent-and-contribution-statement-bib-25-2562_r1_bbag279.pdf]

Author Consent and Contribution Statement

Manuscript Title: DPF: Integrating Functional Specificity with General Plausibility for Targeted Protein Evolution

Manuscript ID: BIB-25-2562.R1

Journal: Briefings In Bioinformatics

Details of Changes to Authorship List and Rationale for the Changes (including the names of authors added/removed and their contributions):

The original authorship list was: Senxin Zhang, Yining Qin, Hanwen Zhu, Feilong Meng, Xiaoqi Zheng.

The revised authorship list is: Senxin Zhang, Yining Qin, Hanwen Zhu, Feilong Meng, Lei Jia, Xiaoqi Zheng.

We have updated the authorship list to include Dr. Lei Jia as a co-corresponding author. Dr. Jia provided essential contributions to the research execution, data processing, and manuscript refinement, fully satisfying authorship requirements. All authors have collectively agreed to this addition and designation.

We, the undersigned, confirm that:

1. We have each made substantial, direct, and intellectual contributions to the work, as stated below.
2. We have reviewed and approved the final version of the manuscript submitted to Briefings in Bioinformatics.
3. We consent to our inclusion as authors and accept accountability for the integrity and accuracy of this research.
4. Our contributions to the manuscript are outlined below using the CRediT taxonomy:

Author Contributions (Please include all co-authors)

Author (please include email address)	Contributions
Senxin Zhang (sxzhang997@qq.com)	Designed the study, conceived the algorithm and analyzed the data
Yining Qin (qinyining2020@sibcb.ac.cn)	Performed assays and analyzed the data
Hanwen Zhu (hanwenzhu998@163.com)	Conceived the algorithm
Feilong Meng (feilong.meng@sibcb.ac.cn)	Designed the study, supervised the research

Lei Jia (jial@sibet.ac.cn)	Conducted the research, performed data analysis, and contributed to the refinement of the manuscript
Xiaoqi Zheng (xqzheng@shsmu.edu.cn)	Designed the study, supervised the research, and drafted the manuscript.

Further details (if needed):

We confirm that all authors listed above have agreed to their inclusion and the accuracy of this authorship statement. This can be confirmed by reaching out to them via their email addresses.

Signatures (In the order in which they appear on the manuscript)

**Author Name**	**Signature**	**Date**
Senxin Zhang	Senxin Zhang	March 18, 2026
Yining Qin	Yining Qin	March 19, 2026
Hanwen Zhu	Hanwen Zhu	March 19, 2026
Feilong Meng	Feilong Meng	March 19, 2026
Lei Jia	Lei Jia	March 18, 2026
Xiaoqi Zheng	Xiaoqi Zheng	March 18, 2026
